# Learning the Concept of Function With Dynamic Visualizations

**DOI:** 10.3389/fpsyg.2020.00693

**Published:** 2020-04-30

**Authors:** Tobias Rolfes, Jürgen Roth, Wolfgang Schnotz

**Affiliations:** ^1^IPN – Leibniz Institute for Science and Mathematics Education, Kiel, Germany; ^2^Institute for Mathematics, University of Koblenz−Landau, Landau, Germany; ^3^General and Educational Psychology, University of Koblenz−Landau, Landau, Germany

**Keywords:** concept of function, covariation, dyna-linking, animation, dynamic visualization, static representation, visual-spatial ability

## Abstract

In this paper we present a laboratory experiment in which 157 secondary-school students learned the concept of function with either static representations or dynamic visualizations. We used two different versions of dynamic visualization in order to evaluate whether interactivity had an impact on learning outcome. In the group learning with a linear dynamic visualization, the students could only start an animation and run it from the beginning to the end. In the group using an interactive dynamic visualization, the students controlled the flow of the dynamic visualization with their mouse. This resulted in students learning significantly better with dynamic visualizations than with static representations. However, there was no significant difference in learning with linear or interactive dynamic visualizations. Nor did we observe an aptitude–treatment interaction between visual-spatial ability and learning with either dynamic visualizations or static representations.

## Introduction

Students in the fields of science, technology, engineering, and mathematics (STEM) often have to acquire knowledge about a process, i.e., a situation that changes over time. In biology, the dynamic process of cell division is key content; in geography, the eruption of a volcano is a process of change over time; in engineering, comprehending how a machine works involves understanding a dynamic situation; and in mathematics, functional relationships (e.g., the path–time relationship of a moving car) often have to be interpreted dynamically—for example, how much does the dependent variable *y* (e.g., path) change if the independent variable *x* (e.g., time) changes by Δ*x*, or at which value of *x* is the strongest increase of *y*?

### Concept of Function

This kind of dynamic thinking is subsumed in mathematics education under thinking of function as *covariation* in contrast to thinking of function as *correspondence* (Vollrath, 1989; Confrey and Smith, 1994; Thompson, 1994). The aspect of correspondence focuses on the pairwise assignment of values of the domain to values of the range. Calculating the function value of a given function (e.g., *f*(*x*) = 2*x*^2^ + 3*x* + 1) for a particular value (e.g., *x* = 5) or finding the zeros of the function *f* are typically function tasks that address the correspondence conception of function. Traditionally, this static view of a function as pointwise relations plays an important role in teaching the concept of function in school (Hoffkamp, 2011; Thompson and Carlson, 2017). The covariation conception, however, focuses on the interdependent covariation of two quantities, that is, the effect of a change of the value of the domain on the value of the range or vice versa. This thinking of function as covariation is considered “fundamental to students’ mathematical development” (Thompson and Carlson, 2017, p. 423). Furthermore, the aspect of covariation is a central aspect of calculus and can, therefore, be considered calculus-propaedeutic. Covariational thinking can be further split into quantitative and qualitative covariation (Rolfes et al., 2018). In a quantitative covariational analysis, a function is examined in numbers (e.g., calculation of a rate of change). In contrast, in a qualitative covariational analysis, the functional relationship is explored by the visual shape of the graph and without the precise function values (Rolfes et al., 2018). Quantitative covariational thinking requires different skills than qualitative covariational thinking, and they form psychometrically two correlated but separate dimensions (Rolfes, 2018).

In mathematics education, one notes that mathematical objects (e.g., functions) are not directly accessible apart from external representations (Duval, 2006). Therefore, a difference exists between the abstract mathematical object and its representations. Hence, a form of representation is needed to deal with a function. The tabular, graphical, algebraic, and situational representation are four typical forms of representation of a function (Janvier, 1978). The unanimous opinion in mathematics education states that the ability to translate between different forms of representations is one aspect of a deep understanding of the concept of function (e.g., Janvier, 1978; Duval, 2006).

### Learning Dynamic Processes With Dynamic Visualizations

One main challenge for teachers and students of all STEM subjects is as follows: how is a dynamic process best learned, and how can we enable students to construct mental models (Johnson-Laird, 1980) that adequately represent the dynamic of the content? The traditional approach uses one or several static pictures to illustrate the process. In textbooks, the cell division process is displayed with static pictures marking crucial steps in the process. Likewise, the process of an eruption of a volcano or the working of a machine is often illustrated with one or more pictures. On the basis of these static pictures, students are required to generate a dynamic mental representation of the processes of cell division, an eruption of a volcano, or the working of a machine. In mathematics, the presentation and learning of dynamic content is even more complicated than in other STEM subjects. If the functional relationship under consideration models a real-life situation (e.g., a path–time relationship), the underlying dynamic situation (e.g., the movement of a car) is often not illustrated at all. Instead, an abstract graph is displayed as a static representation of the functional relationship. Students are required to draw a connection between the real-life situation and the underlying functional relationship on the basis of this static graph. Afterward, they have to “animate” the graph mentally to solve a covariation task (e.g., does the speed of the car increase or decrease?). With the advent of modern technology, a new approach to learning dynamic content has become possible: *dynamic visualizations* (e.g., animations) of processes (e.g., cell division, eruption of a volcano) that can display the dynamic content dynamically. This approach corresponds with the notion held by many that static representations are the best method for learning about static content, and dynamic visualizations the most appropriate for dynamic content (Ploetzner and Lowe, 2004; Schnotz and Lowe, 2008). Based on this congruency hypothesis between external and mental representations, for example, Karadag and McDougall (2011) argued that e.g., “the term ‘increasing’ points out a dynamic process, which is quite difficult to understand in a static media” (p. 175).

Dynamic visualizations can be defined as representations that change their graphical structure during the presentation (Schnotz et al., 1999; Ploetzner and Lowe, 2004). Kaput (1992) considered as characteristic for dynamic visualizations that time has an “information-carrying dimension” (p. 525). In dynamic visualizations, the states of objects can change as a function of time (Kaput, 1992). Dynamic visualizations can be further subdivided into *linear dynamic* and *interactive dynamic* visualizations. In the case of linear dynamic visualizations (e.g., non-interactive animations), the change takes place automatically and cannot be influenced. Interactive dynamic visualizations, on the other hand, give learners “some control over how these changes are presented to them” (Ploetzner and Lowe, 2004, p. 235). Schwan and Riempp (2004) pointed out that interactive dynamic visualizations “enable the user to adapt the presentation to her or his individual cognitive needs” (p. 296). However, interactivity could also have negative effects on cognition if managing interactive features burdens the learner with additional cognitive load (Schwan and Riempp, 2004).

For scientific content, empirical findings concerning learning with dynamic visualizations could seldom corroborate assumed advantages for this mode of learning. Often, dynamic visualizations showed no higher learning effect than static representations. In an experiment conducted by Hegarty et al. (2003), understanding of how flushing cisterns work increased when both static representations and dynamic visualizations were used; however, there was no evidence that dynamic visualizations led to a higher learning effect than did static representations. Mayer et al. (2005) found no advantages in instructions containing dynamic visualizations regarding learning about various types of scientific content (braking systems, ocean waves, toilet tanks, lightning). Instead, for some content, learning with paper-based static representations proved significantly more effective than learning with dynamic visualizations.

### Dyna-Linking as a Form of Dynamic Visualization in Mathematics

In mathematics, a graph is a pivotal form of representation when dealing with the concept of function. The ability to connect the situational with the graphical representation is considered essential to understanding graphs (Janvier, 1978; Hoffkamp, 2011). One approach to foster this ability is providing a real-time link between a motion and a graphical representation (e.g., Brasell, 1987; Thornton and Sokoloff, 1990; Nemirovsky et al., 1998; Radford, 2009; Urban-Woldron, 2014). This real-time link can be produced by motion detectors that record motions of persons or objects with a sensor. These data are then displayed in real-time as a kinematic Cartesian graph on a screen, and students have to explore and interpret these kinematics graphs. Brasell (1987) found out in an experiment that the immediate display of the graph on a screen is crucial since a lag of only 30 s already impaired learning. Nemirovsky et al. (1998) concluded, based on their case study with a motion detector, that graphing motions “allows students to encounter ideas such as distance, speed, time, and acceleration” (p. 169). The learning environments using a motion detector have in common that they try to foster a rather conceptual and qualitative than a procedural and quantitative understanding of functional relationships.

**FIGURE 1:**
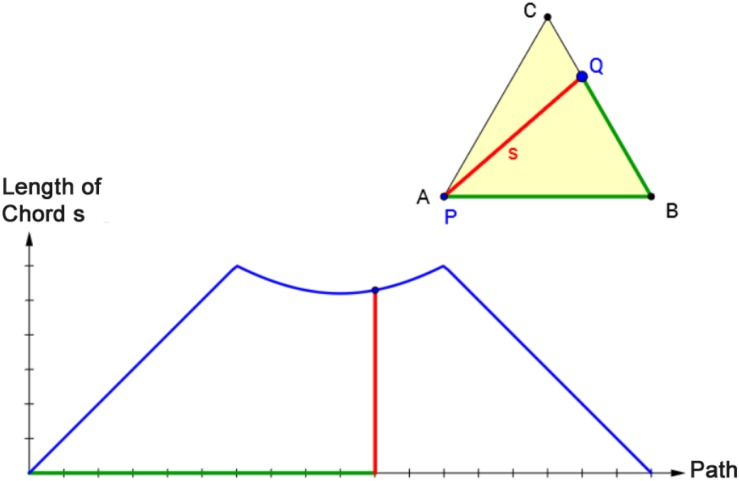
Screenshot (translated into English) of dyna-linking two representations (equilateral triangle and corresponding graph). The effect of a movement of point Q is displayed simultaneously in the triangle ABC and the coordinate system.

A related approach to highlight the connection between two forms of representation is through dynamic linking of representations in a dynamic visualization; this is referred to as *hot linkages* (Kaput, 1992) or *dyna-linking* (Ainsworth, 1999). In dyna-linking, two representations are linked so that the effect of an action in one is automatically displayed in the linked second (Kaput, 1992; Ainsworth, 1999). Figure 1 shows an example of dyna-linking a representation of an equilateral triangle with a graph. The graph displays the relationship between the length of the path on the perimeter of triangle ABC from P to Q and the length of the corresponding chord PQ. Starting in vertex A, point Q moves counterclockwise along the triangle line until it reaches vertex A again. The effect of this alteration is simultaneously displayed in the triangle and the graph.

In educational research, various reasons for the advantageous nature of dyna-linking were put forward. One says a system that automatically translates between forms of representation should reduce learners’ cognitive load, thereby freeing up cognitive capacity to learn the relationship between representations (Kaput, 1992; Scaife and Rogers, 1996; Ainsworth, 1999; Karadag and McDougall, 2011). Some researchers also considered the idea of supplantation (Salomon, 1979/1994) as the underlying beneficial principle of dyna-linking (Vogel et al., 2007; Hoffkamp, 2011). Salomon postulated that mental operations could supplant mental operations if learners are unable to perform the operations by themselves. Vogel et al. (2007) pointed out that supplantation can support the learner’s mental operations in connecting a graph with the underlying situation concerning both aspects of a function (correspondence and covariation). Furthermore, the framework of *instrumental genesis* (Rabardel, 2002) can be considered as a theoretical underpinning of the effectiveness of dyna-linking. When a dynamic visualization in the form of dyna-linked representations as an *artifact* is put into an interactive relationship with a specific task and students’ mental schemes, it transforms into an *instrument* that can enhance learning.

Some empirical studies evaluated the effect of dynamic visualizations in terms of dyna-linking situational and graphical representations on learning covariational aspects of the concept of function. Hoffkamp (2011) performed a qualitative study with 25 10th grade students. A geometrical situation (area within a triangle) was dynamically linked to the corresponding graph (relationship between area and a length) in a learning environment. Hoffkamp concluded that dyna-linked interactive visualizations “not just lead to the manipulation of some points or lines, but really activate the formation of an intuitive access of calculus” (p. 370). She observed that especially asking for verbalizations prompted conceptualization processes and led to students integrating a dynamic view into their conception of function (Hoffkamp, 2011).

In an experimental study with 133 middle-school students, Vogel et al. (2007) evaluated the effect of supplantation on the ability to interpret graphs. The students were divided into three experimental groups. The *full supplantation* group had to interpret graphs concerning variables of a geometric object (e.g., relationship between radius and surface area of a cylinder when the volume is fixed). They received support via an interactive dynamic visualization that dyna-linked the graph with a representation of the geometric object. In the *reduced supplantation* group, the graph was linked with a representation of the geometric object for one particular value, but no dyna-linking was available. In the *no-supplantation* group, the students only had the graph available and no representation of the geometric object at all. The experiment showed that linking the graph with a representation of the geometric object had a significantly positive effect on learning to interpret graphs. There was, however, no significant difference between the two forms of linking (full vs. reduced supplantation), that is, dyna-linking was not more beneficial than linking the graphical and situational representations in a static manner.

In two experiments with 111 eleventh graders and 24 tenth graders, Ploetzner et al. (2009) investigated which kind of visualizations most helped students to relate motion phenomena to line graphs. The students in the control group only received dyna-linked representations of a moving runner and the corresponding piecewise line graph. In the experimental group, the students also received vectors representing the distance covered by the runner at different points in time. The result showed that adding vectors which dynamically represent the covered distance compared to “only” dyna-linking the motion of the runner with the piecewise line graph had no additional effect.

### What Are Favorable Conditions for Learning With Dynamic Visualizations?

Lowe and Ploetzner (2017) conclude that dynamic visualizations have “not proven to be the educational magic bullet that many assumed it would” (p. xv). The explanation for the rather disappointing empirical results concerning learning with dynamic visualizations remains up for discussion. van Gog et al. (2009) suggest that dynamic visualizations place a higher load on working memory; that is, learners need to process the information that is visible at the time as well as remember previous information, and relate and integrate that information to understand the dynamic visualization. These requirements, combined with a constant stream of information, increase the load on working memory. As a result, information shown at the beginning of a dynamic visualization might be lost from memory before it can be linked to information shown later. These problems of transitivity do not exist with static representations because they can be studied repeatedly (van Gog et al., 2009; Höffler and Leutner, 2011). Additionally, from a constructivist perspective, dynamic visualizations, like dyna-linking, can be considered problematic because learners may remain too passive or even be discouraged from worrying about translations of representations (Ainsworth, 1999). This could result in the desired ability to perform translations between representations not being developed by dyna-linking (Ainsworth, 1999). Mayer et al. (2005) have speculated that the mental simulation of a dynamic process based on a static representation could achieve a higher learning effect than that achieved by merely receptively contemplating a dynamic visualization.

The lack of solid empirical evidence for a learning effect of dynamic visualizations, combined with various theoretical rationales concerning the disadvantages, raises the question of whether there are any circumstances in which dynamic visualizations are conducive to learning. Pea (1985) gave some fundamental thoughts on the role of computers and dynamic visualizations. He argued that the computer could be viewed as cognitive technology that not only amplified but reorganized cognition and “helps transcend the limitations of the mind” (p. 168). Therefore, in mathematics, the use of computers and dynamic visualizations shifts the activities more to a meta-level (e.g., interpreting graphs instead of constructing graphs from a table) instead of doing the same as before but “faster, more often and more accurately” (Dörfler, 1993, p. 168). As a consequence, new kinds of tasks are necessary to initiate cognitive activities on the meta-level (Dörfler, 1993).

Some researchers tried to identify the functional role of dynamic visualizations in learning a given content. Schnotz and Rasch (2008) proposed that dynamic visualizations could promote learning if cognitive resources are freed up: if a mental process becomes feasible for a learner only through dynamic visualization, it fulfills an *enabling* function. If a process can also be carried out with the aid of a static representation, but the dynamic visualization considerably reduces an otherwise very high cognitive load, the dynamic visualization has a *facilitating* function (Schnotz and Rasch, 2008). Consequently, dynamic visualizations should be most effective in challenging tasks. Tversky et al. (2002) suggested a congruence principle between external and internal representations: dynamic visualizations are only more beneficial than static representations when the dynamically presented content is congruent with the internal representations that the learner must construct.

Furthermore, some general conditions appear to influence learning with dynamic visualizations positively. First, *interaction* options while learning with dynamic visualizations appear to enhance learning. Experiments have shown that even relatively small interactive elements, such as pausing and replaying a dynamic visualization, can increase learning success (e.g., Mayer and Chandler, 2001; Hasler et al., 2007). This positive effect could be caused by the reduction of cognitive burden on working memory (Spanjers et al., 2010). In general, interactively manipulating dynamic visualizations could enhance learning because they hinder the acceptance of a dynamic visualization in a passive way (De Koning and Tabbers, 2011). Nevertheless, even interaction options are Janus-faced: they can also produce negative effects, such as random clicks or the omission of interaction options (De Koning and Tabbers, 2011). Interactive information places additional demands on learners and potentially limits the cognitive resources available, thus detrimentally affecting the learning process. One could reduce the processing demands of interactivity by constraining the experiment space in an interactive dynamic visualization (Klahr and Dunbar, 1988; van Joolingen and de Jong, 1997), that is, reducing the interaction possibilities.

Second, *cognitive activation* appears essential when learning with dynamic visualizations. Hegarty et al. (2003) found that understanding increased when learners had to predict the dynamic behavior of a machine from static representations. De Koning and Tabbers (2011) concluded that interactive manipulations combined with understanding processes might increase the learning effect of dynamic visualizations. Additionally, De Koning et al. (2009) advocated highlighting certain parts of a dynamic visualization in order to draw learners’ attention to these areas.

### The Role of Visual-Spatial Ability in Learning With Dynamic Visualizations

In addition to general factors like interaction and cognitive activation that appear to enhance the learning effect, moderating factors might influence the impact of dynamic visualizations on learning. Dealing with dynamic visualizations requires visual-spatial ability. Therefore, visual-spatial ability could have a moderating effect on learning with dynamic visualizations, thereby generating an aptitude–treatment interaction (Snow, 1989). In the literature, there are two competing theses about the aptitude–treatment interaction between visual-spatial ability and learning with dynamic visualizations. On the one hand, the *ability-as-compensator* hypothesis assumes that dynamic visualizations are particularly advantageous for learners with low visual-spatial ability (Mayer and Sims, 1994; Mayer, 2001). People with low visual-spatial ability are less able to animate their own mental representations and use dynamic visualizations to compensate for their lack of skill (Hegarty and Kriz, 2008; Höffler and Leutner, 2011; Sanchez and Wiley, 2014). Therefore, the availability of external dynamic visualizations could help learners with limited spatial imagination to construct satisfactory mental models (Hegarty and Kriz, 2008; Höffler and Leutner, 2011), the dynamic visualization serving as a “cognitive prosthesis” (Hegarty and Kriz, 2008, p. 7). Further, a theoretical foundation for the compensation thesis can be deduced from the theory of supplantation (Salomon, 1979/1994). For our research, the theory of supplantation would imply that external dynamic visualizations could supplant mental processes related to dealing with functional relationships requiring visual-spatial imagination.

The *ability-as-enhancer* thesis, on the other hand, assumes that learners with good spatial imagination benefit more from dynamic visualizations than do learners with poor spatial imagination (Mayer and Sims, 1994; Huk, 2006; Höffler and Leutner, 2011). In this case, visual-spatial ability serves to amplify the learning process. An amplifying effect could result because dynamic visualizations may place a higher demand on spatial imagination due to their transitivity than static representations (Höffler and Leutner, 2011). Thus, only students with high visual-spatial ability would be able to process the information presented in rapid succession in a dynamic visualization (Hegarty and Kriz, 2008), because visual-spatial imagination is associated with larger spatial working memory (Miyake et al., 2001). This relationship would make dynamic visualizations detrimental to learners with poor visual-spatial ability.

Empirical results on the aptitude–treatment interaction between visual-spatial perception and learning with dynamic visualizations are inconsistent. In an experiment with 162 students, Sanchez and Wiley (2014) found no aptitude–treatment interaction between the performance in a paper folding task and the learning with dynamic visualizations. In three experimental groups, the students had to read a text about the eruption process of a volcano. The text was accompanied either by static pictures or by a linear dynamic visualization or there were no pictures at all. Nevertheless, in the same study but using another measure of visual-spatial ability—a test for predicting the motion of various objects—dynamic visualizations were found to have a compensating effect (Sanchez and Wiley, 2014). Narayanan and Hegarty (2002) and Hegarty et al. (2003) failed to find an aptitude–treatment interaction in an experiment using static illustrations and non-interactive animations with 100 students learning how a flushing cistern works. Höffler and Leutner (2011), on the other hand, identified a compensating effect of dynamic visualizations in an experiment examining chemical content (role of surfactants during the washing process) involving 25 students. The text was illustrated either with a system-paced animation or four static pictures representing the key moments of the process. In a second experiment with 43 students, these same authors (Höffler and Leutner, 2011) were able to replicate an aptitude–treatment interaction.

### Present Study

The theoretical findings raise the question to what extent dynamic visualizations influence learning of a core mathematical idea like the concept of function. Therefore, the present study investigated the following three hypotheses:

Hypothesis 1 (H1): Dynamic visualizations of geometrical situations dyna-linked with the corresponding graph are more beneficial than only providing static representations of a geometrical situation and the corresponding graph for learning about the aspect of covariation of a function.

Learning with dynamic visualizations is not more beneficial *per se* than learning with static representations. Dealing with functional relationships that focus on the aspect of covariation does, however, require the execution of dynamic mental processes. A higher learning effect of dynamic visualizations compared with static representations is to be expected if dynamic visualizations considerably facilitate the learning process, or even just enable it (Schnotz and Rasch, 2008).

Hypothesis 2 (H2): Using interactive dynamic visualizations of geometrical situations dyna-linked with the corresponding graphs are more beneficial than using linear dynamic visualizations for learning about the aspect of covariation of a function.

Interactive dynamic visualizations allow or even require learners to influence the flow of a dynamic visualization. Therefore, learners can control the flow of information and prevent the information overload of working memory. In addition, systematic variations can be deliberately explored. However, the number of variations in the interactive dynamic visualization should be kept low to facilitate a focused learning process.

Hypothesis 3 (H3): There is an aptitude–treatment interaction between visual-spatial ability and learning about the aspect of covariation of a function with linear or interactive dynamic visualizations of geometrical situations dyna-linked with graphs.

The ability-as-compensator and the ability-as-enabler hypotheses offer two rationales postulating an aptitude–treatment interaction between visual-spatial ability and representational form, albeit in different directions.

## Materials and Methods

### Overview and Experimental Design

**FIGURE 2:**
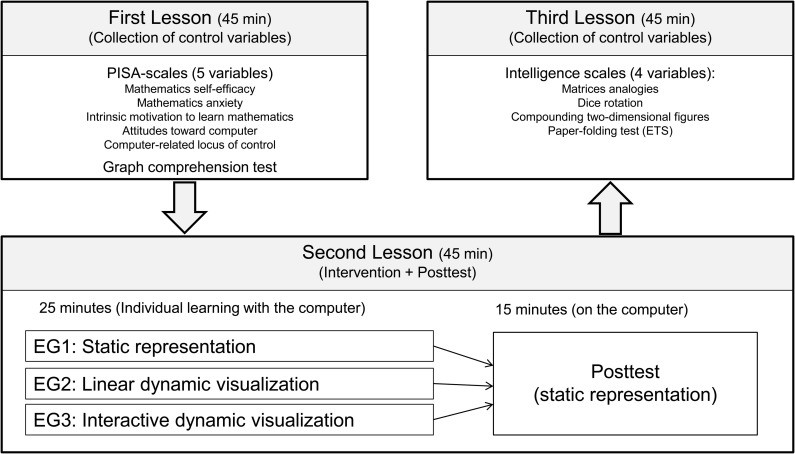
Overview of the experimental design.

An experiment consisting of three lessons of 45 min each was performed to check the validity of the hypothesis (cf. Overview in Figure 2). In the first lesson, six control variables were collected (cf. subsection instruments). The intervention with a computer-based learning environment took place in the second lesson (cf. subsection learning environment). The students were randomly assigned to one of three experimental groups and individually learned for 25 min using a static representation, a linear dynamic visualization, or an interactive dynamic visualization. The core content of the learning environment was the learning of qualitative covariational thinking. The computer-based posttest was administered during the second lesson, immediately following the intervention. Finally, four more control variables were collected in the third lesson. The whole experiment took place in three mathematics lessons within one school week.

### Participants

One hundred and fifty-seven students (88 eighth-graders; 69 ninth-graders) of an academic track secondary school (Gymnasium) in the German state of Rhineland-Palatinate participated in the study. Nearly all students of the seven Grade 8 and 9 classes voluntarily participated in the experiment. Each gender was almost equally represented (55% female; 42% male; 3% N/A). The mean age was 14.2 years (SD = 0.66). The state’s curriculum requires functional relationships to be covered in grades 8 and 9 (Ministerium für Bildung et al., 2007). The focus of the curriculum, however, is on linear and quadratic functions, the procedural-technical handling of algebraic expressions, and the display in graphs. A qualitative analysis of general functional relationships, in particular with regard to the aspect of covariation, is not a regular part of mathematics lessons in these grades. Therefore, the content of the intervention and the posttest (see below) can be considered relatively unknown to the students.

### Learning Environment

**FIGURE 3:**
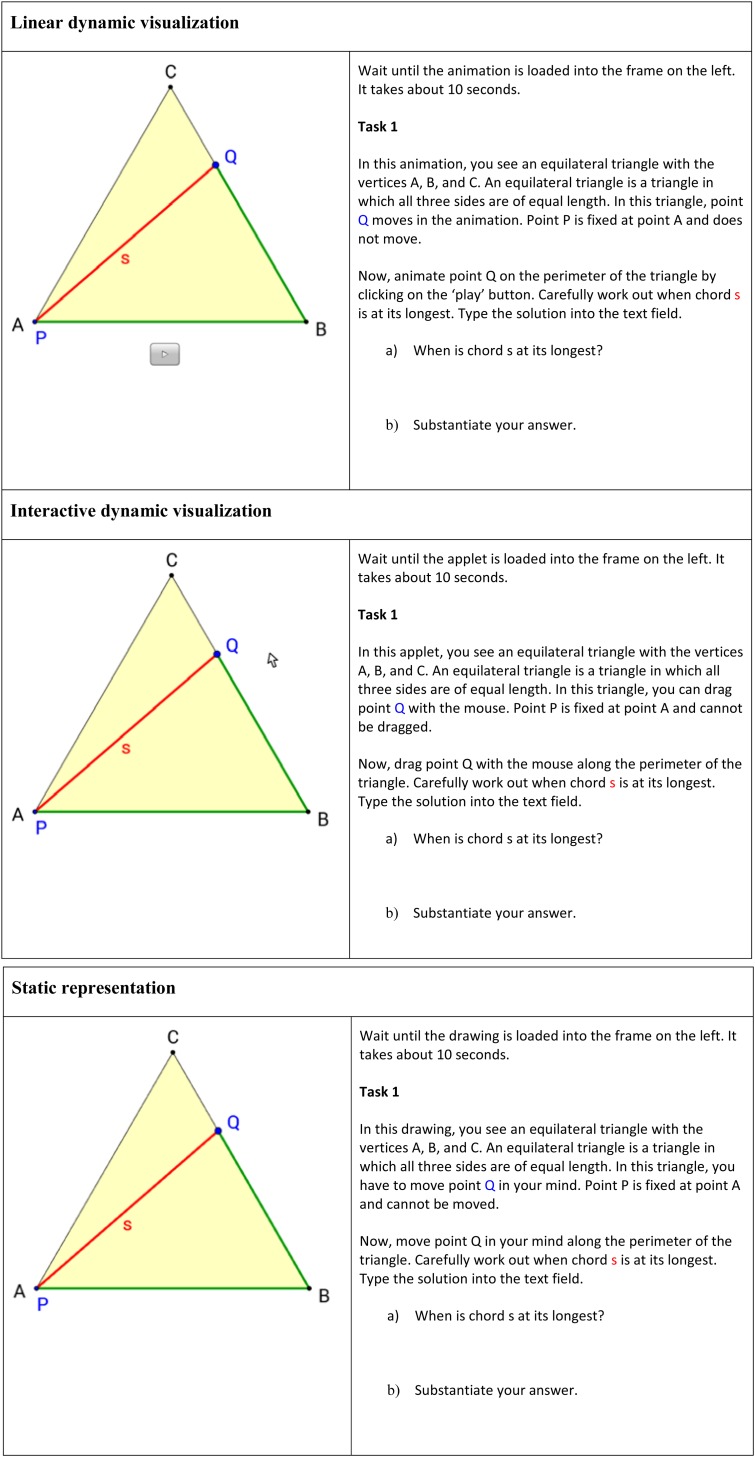
First task of the three experimental conditions in the learning environment (translated into English).

The computer-based learning environment consisted of 19 tasks. The aim of the learning environment was to foster students’ ability in qualitative covariational thinking. The stimulus in the first task (Figure 3) was an equilateral triangle, in which a chord was drawn from point P to a point Q. The chord’s endpoint Q was variable, while the starting point P was fixed at vertex A. Thus, this geometric configuration constituted a functional relationship between the length of the path on the perimeter of triangle ABC from point A to point Q and the length s of the chord PQ. We selected this problem as the initial content of our learning environment because it provided different demanding covariational tasks (cf. Roth, 2005) and was almost certainly unknown to the students. This geometrical configuration required students to evaluate what effect a variation of the geometrical configuration, that is, moving the endpoint of a chord, has with regard to covariational aspects. Intentionally, quantitative covariational thinking was not addressed. Instead, the focus was to prompt a more conceptional understanding of covariation.

In Task 1, the students had to work out at which point the chord was at its longest based on the representation of the equilateral triangle. The students had to substantiate their answer to stimulate cognitive activation and to avoid guessing behavior. In Task 2, the students had to argue at which point the chord was at its shortest. The same representation of an equilateral triangle was also used in the following tasks 3 to 6, in which students were asked further questions about the functional relationship between the length of the path and the length of the chord—for example, in which part does the length of the chord increase, and in which part does it decrease? The intention of the first six tasks was to engage students in covariational thinking in a geometrical situation. The graph was purposely not introduced before this point. Rather, the students should first acquire a profound understanding of the situational context and its covariational aspects.

Students learned the connection between the situational and graphical representation in the following six tasks according to a predict-observe-explain scheme that has shown beneficial in previous research (Urban-Woldron, 2014). In tasks 7 to 9, students had to predict the form of the graph for different sections (point Q moving from A to B, from B to C, and from C to A). Corresponding lengths were colored with the same color (Kozma, 2003) to support the students’ ability to translate between the situational and graphical representation. Tasks 10 to 13 displayed the connection between the representation of the triangle and the complete graph of the functional relationship (c.f., Figure 1) so that students could check the correctness of their predictions and explain why the graph has this particular form. In the last six tasks (tasks 14 to 19), students had to answer similar questions for a rectangle instead of a triangle. None of the students’ answers were assessed or explicitly corrected.

The tasks of three experimental groups were accompanied by three different forms of representation in the learning environment (Figure 3): when learning with the linear dynamic visualization, students could only watch an animation and observe the movement of a point Q on the triangle line ABC and its effect on the length of chord PQ; the students learning with the interactive dynamic visualization could use their mouse to drag the point Q along the triangle line and study the effect of their manual manipulation; students in the third experimental group had to solve the same tasks using static representations and to simulate mentally the point’s movement without external support.

The mathematical content of the 19 tasks in the learning environment was identical for the three experimental groups, but the instructional text differed where necessary. For example, students using a linear dynamic visualization were instructed to “animate point Q on the perimeter of the triangle by clicking on the play button.” Those working with an interactive dynamic visualization were asked to “drag point Q with the mouse along the perimeter of the triangle,” while those using a static representation were prompted to “move point Q in your mind along the perimeter of the triangle.”

During the intervention, every student worked with the digital learning environment without external support of instructors. Collaboration between students was not allowed and did not take place. The students were unaware of the experimental variation and which group they belonged to until the very end of the experiment.

The original German learning environment is reported in Supplementary Material 1.

### Instruments

A number of variables were collected on participants’ attitudes and abilities. The main reason for including these variables was to check whether the randomized assignment into experimental groups led to groups with approximately equivalent preconditions. Furthermore, these variables allow controlling their effect on the outcome (cp. Maxwell et al., 2018). Therefore, we tried to identify covariates that could be assumed to correlate with the posttest (see explanation below) as the outcome variable from a theoretical or empirical perspective. We selected the three scales for measuring *mathematics self-efficacy*, *mathematics anxiety*, and *intrinsic motivation to learn mathematics* (Ramm et al., 2006) from the program for international student assessment (PISA). We specifically chose these because, as our own secondary analysis of PISA data showed, they displayed substantial predictive power for mathematics performance in the German PISA 2003 sample. In addition, we included the two PISA variables *attitudes toward computers* and *computer-related locus of control*, because of the computer-based learning setting of our experiment. Cognitive potential usually has high predictive power on mathematics performance. Therefore, we administered the subtest *matrices analogies* in the German adaptation of the *cognitive ability test* (Heller and Perleth, 2000). Additionally, visual-spatial ability was assessed because it is a relevant part of intelligence and because we assumed an ATI-effect between visual-spatial ability and learning with dynamic visualization (cp. H3). We used three different scales: the first, *dice rotation*, and second, *compounding two-dimensional figures*, were selected from the German intelligence test I-S-T 2000R (Amthauer et al., 2001); the third was the *paper-folding test* of the Educational Testing Service (Ekstrom et al., 1976). In general, a further important predictor of mathematics performance is prior knowledge. Because the learning environment and the posttest included graphs, we assessed students’ ability to deal with graphs. Hence, we developed a *graph comprehension test* that had sufficient internal consistency (α = 0.73). It consisted of 21 items that required students to analyze graphs qualitatively. The original German graph comprehension test is presented in Supplementary Material 2.

**FIGURE 4:**
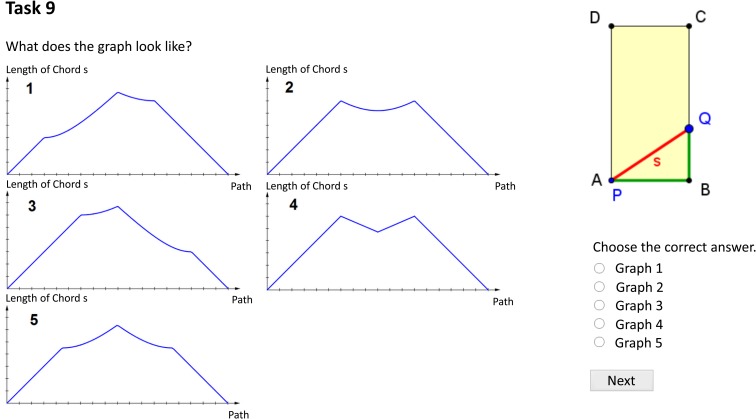
Exemplary item of the posttest (translated into English).

The computer-based posttest (α = 0.71) comprised 14 items (see Figure 4). Here, students had to apply or “transfer” their acquired knowledge to different figures (e.g., rectangular triangles, rectangles, pentagons). Static representations accompanied all the items because we were interested in how dynamic visualizations can improve the learning process and prompt elaborate mental representations so that the students can subsequently apply their acquired knowledge on static representations without the need for dynamic visualizations. As Dörfler (1993) pointed out, “so-called visualizations of mathematical concepts […] remain an integrative and constitutive part of the respective concept for the individual” (p. 169). The posttest was designed as a level test, and the students were given sufficient time (approx. 15 minutes) to complete all the items. The original German posttest items are reported in Supplementary Material 3.

### Posttest-Only Design

We used a posttest-only design for the following reasons. First, because students were randomly assigned to one of the three experimental conditions, and the group sizes were sufficiently large. Hence, we can assume that confounding variables (e.g., prior knowledge, intelligence) are balanced out in the groups (Maxwell et al., 2018). Second, we feared an interaction between pretesting and the intervention, that is, that the students would behave differently with a pretest, because of the specific nature of the learning content. Third, we collected several covariates to control for the effect of these variables on the outcome. Fourth and finally, in a pre-posttest-design, there is a risk of the test showing a floor effect in the pretest or a ceiling effect in the posttest. Therefore, we decided the best way to perform the experiment was to refrain from administering a content-specific pretest.

### Data Analysis

#### Analysis of the Experimental Effect

A covariance analysis was performed to analyze whether the learning effects in the three experimental groups differed significantly. The advantage of a covariance analysis over an ANOVA is that it additionally takes into account the effect of the control variables on the outcome (Field et al., 2012; Tabachnick and Fidell, 2014). We used the regression approach of a covariance analysis because it leads to identical results as an ANCOVA but is more general and flexible (Field et al., 2012; Tabachnick and Fidell, 2014).

The first hierarchical regression analysis was intended to identify covariates that had a significant impact on the posttest. Therefore, the control variables were gradually added to the model as predictors in a first regression model. The order of entry to the model was based on theoretical expectations of which variables might explain a larger proportion of the variance. Significant predictors for the posttest were ultimately identified as covariates based on the results of the hierarchical regression analysis.

The covariance analysis was performed in the second regression analysis. Orthogonal contrasts were used to determine the experimental effect. Since the design of the experiment was slightly unbalanced due to randomization—that is, the three experimental groups did not have the exact same number of subjects—the contrast coefficients had to be adjusted to ensure the orthogonality of the contrasts (c.f., Pedhazur, 1997). A total score for visual-spatial ability was generated by calculating a mean of the standardized values of the three different visual-spatial ability variables.

#### Analysis of the Aptitude–Treatment Interaction

A moderated regression analysis was performed to analyze the aptitude–treatment interaction between visual-spatial ability and experimental effect.

#### Dealing With Missing Values

Items not seen by a student due to absence during the experiment were coded as missing. Items on the ability scales seen but not answered by students were rated as incorrect (graph comprehension, posttest, matrices analogies, dice rotation, compounding two-dimensional figures, and paper-folding test). In the case of the attitude scales (mathematics self-efficacy, mathematics anxiety, intrinsic motivation to learn mathematics, attitudes toward computers, and computer-related locus of control), seen but unanswered items were coded as missing.

Of the 157 students, five were not present for all three lessons of the experiment. As a result, several of their scale values were incomplete. Therefore, the data of these five subjects were excluded from the analysis. Of the 152 students who participated in all three lessons, six had at least one missing value on an attitude scale because they had not answered one or more items. Therefore, the missing values of these six students were replaced by multiple imputations. Overall, four control variables were affected by the imputations. The regression analyses were therefore performed based on the observed and imputed data of these 152 students.

In the multiple imputations, five imputations were performed resulting in five complete data matrices for the remaining 152 students. Hierarchical regression was performed on each of these five data matrices, and the test statistics pooled. The pooling of the F-values was determined using the $D_{1}^{*}$-statistic (Reiter, 2007), while the pooling of the determinative coefficient R^2^ was performed using Fisher’s (1915) z-transformation (c. f., Enders, 2010). The regression coefficients and their standard errors were calculated in accordance with Rubin’s (1987) approach. For the significance testing of the pooled regression coefficients by t-tests, the adjusted degrees of freedom were determined using Barnard and Rubin’s (1999) formula for small to medium sample sizes.

#### Software

Regression analyses were performed using the software package *R* (R Core Team, 2017). Multiple imputations were calculated with the package *Mice* (van Buuren and Groothuis-Oudshoorn, 2011).

## Results

### Learning Effect of Experimental Groups (H1 and H2)

**FIGURE 5:**
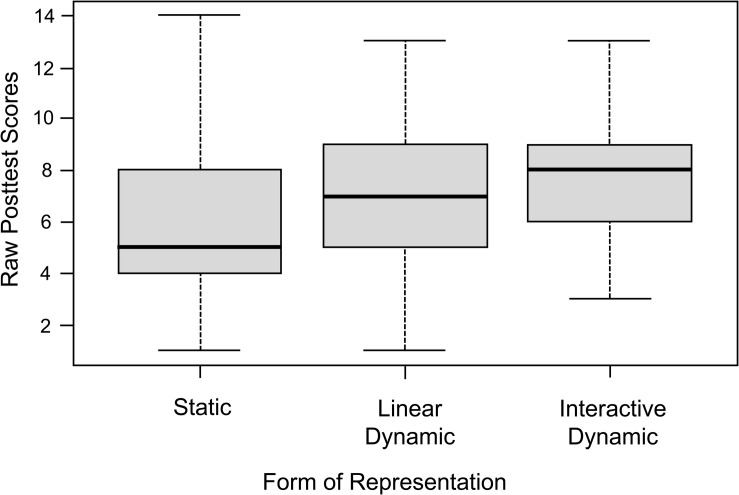
Boxplots of the raw posttest scores of the three experimental groups.

The descriptive analysis of the posttest results showed mean differences between the three experimental groups (see Figure 5). The group learning with static representations had a mean posttest score of M = 5.98 (SD = 3.12), while the groups learning with linear dynamic and interactive dynamic visualizations achieved a mean posttest score of M = 7.04 (SD = 3.04) and M = 7.67 (SD = 2.79), respectively.

To determine whether the means differed significantly, a covariance analysis was performed by inserting covariates as predictors in a multiple regression model. An analysis of variance showed that the mean score of the control variables did not differ significantly between the three experimental groups (see Table 1). In addition, no signs of significant variance heterogeneity were found, as revealed by Levene’s test (see Table 1). Furthermore, different regression weights of the control variables could not be identified. Thus, three important preconditions for covariance analysis (covariate independent of group effect, variance homogeneity, and homogeneous regression weights) could be assumed.

**TABLE 1:** Descriptive statistics of the control variables.

	Experimental group			ANOVA		Levene	
Variable	S *n*_1_ = 54 *M* (*SD*)	LD *n*_2_ = 50 *M* (*SD*)	ID *n*_3_ = 48 *M* (*SD*)	*F*^b^	*p*	*F*^b^	*p*
Graph comprehension	10.1 (3.4)	9.0 (4.1)	10.4 (4.2)	1.65	0.20	1.40	0.25
Dice rotation	9.4 (3.4)	9.5 (3.3)	10.1 (4.0)	0.61	0.54	1.77	0.17
Compounding two-dimensional figures	9.9 (3.5)	10.2 (3.6)	10.7 (3.4)	0.64	0.53	0.09	0.91
Paper-folding test	11.3 (3.8)	10.9 (3.8)	11.9 (3.9)	1.33	0.27	0.09	0.92
Matrices analogies	16.9 (5.8)	15.9 (6.0)	16.8 (5.2)	0.42	0.66	0.43	0.65
Intrinsic motivation to learn mathematics^a^	7.8 (2.7)	8.7 (2.7)	8.6 (2.6)	2.02	0.13	0.51	0.60
Attitudes toward computers	11.7 (2.7)	11.3 (2.7)	11.1 (3.4)	0.65	0.52	2.40	0.09
Computer-related locus of control^a^	21.4 (5.0)	21.5 (5.2)	20.6 (5.4)	0.44	0.64	0.05	0.95
Mathematics anxiety^a^	9.8 (3.6)	9.0 (3.4)	8.6 (3.4)	1.67	0.19	0.45	0.64
Mathematics self-efficacy^a^	23.6 (3.2)	24.0 (3.6)	24.2 (2.9)	0.46	0.63	1.55	0.21

*S = static; LD = linear dynamic; ID = interactive dynamic. ^a^The values for these variables represent pooled values based on five imputations. ^b^Degrees of freedom of the F values of the variables vary depending on whether missing values have been imputed or whether the answers were complete.*

In the first multiple hierarchical regression (see Table 2), significant predictors for the posttest were identified for later inclusion as covariates in the analysis. For this purpose, the graph comprehension test was included in the regression model in step 1. The graph comprehension test had a significant influence, β = 0.47, *t*(151) = 6.58, *p* < 0.001 and explained 22.3 percent of the variance of the posttest score, *F*(1, 151) = 43.31, *p* < 0.001. An additional significant 9.3 percentage point of explained variance was provided by the four different facets of intelligence (matrices analogies, dice rotation, compounding two-dimensional figures, and paper-folding test), *F*(4, 147) = 5.01, *p* < 0.001. The regression weights of the four individual variables did not, however, differ significantly from 0. Including the scales for attitude to mathematics in step 3 significantly increased the proportion of variance explained by a further 7.2 percentage points, *F*(3, 148.03) = 5.62, *p* = 0.001. However, only the regression weight of the variable intrinsic motivation to learn mathematics was significant, β = 0.30, *t*(141.98) = 3.56, *p* < 0.001. In step 4, the scales anxiety in mathematics and self-efficacy in mathematics were also included in the regression model. Here as well, the regression coefficients did not differ significantly from 0; nor did the inclusion of the two variables significantly increase the proportion of variance explained, *F*(2, 149.03) = 1.50, *p* = 0.23.

**TABLE 2:** Hierarchical regression with posttest as dependent variable.

	Posttest score	
Predictor	*ΔR^2^*	ß
**Step 1**	0.223***	
Graph comprehension		0.47***
**Step 2**	0.093***	
Dice rotation		0.12
Compounding two-dimensional figures		0.08
Paper-folding test		0.16
Matrices analogies		0.08
**Step 3**	0.072**	
Intrinsic motivation to learn mathematics		0.30***
Mathematics anxiety		0.06
Mathematics self-efficacy		0.09
**Step 4**	0.013	
Attitudes toward computers		–0.166
Computer-related locus of control		0.12
Total *R*^2^	0.401***	
*N*	152	

From step 3 on, the coefficients of determination and the regression coefficients are pooled values based on five imputations. ** p < 0.01, *** p < 0.001.

In a second step, control variables from the first regression analysis were summarized for or eliminated from inclusion as covariates in a regression model. The four variables measuring cognitive ability (matrices analogies, dice rotation, compounding two-dimensional figures, and paper-folding test) showed multicollinearity from both a theoretical and an empirical point of view. As multicollinearity should be avoided in multiple regression (Tabachnick and Fidell, 2014), the four scales were aggregated into a single value as the standardized sum of the individual variable values. Since all four scales were sub-facets of intelligence tests, this aggregated value was called *intelligence*. Of the five attitude scales, only the intrinsic motivation to learn mathematics variable was used as a covariate in the second regression model since the four other variables did not significantly contribute to the variance explained.

**FIGURE 6:**
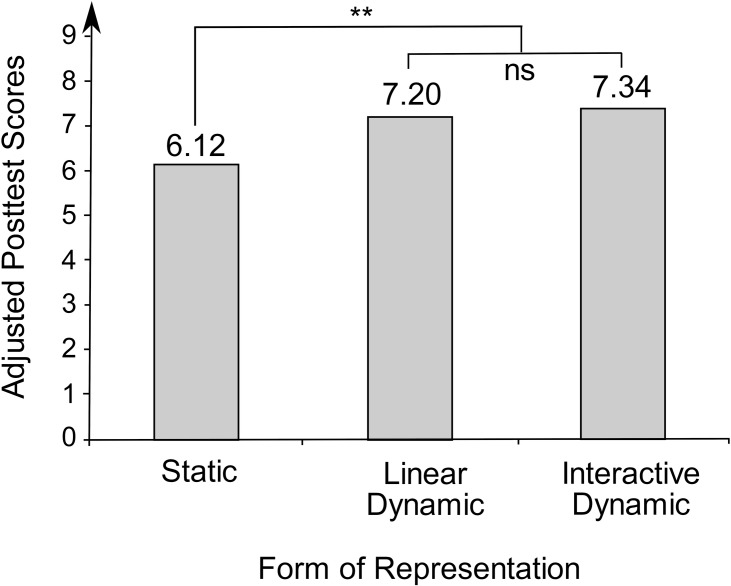
Adjusted mean posttest scores of the three experimental groups. **p < 0.01; ns = nonsignificant.

Thus, the three covariates graph comprehension, intelligence, and intrinsic motivation to learn mathematics were included as predictors in the second hierarchical multiple regression model (see Table 3). Together, they accounted for 38.1 percent of the variance of the posttest, *F*(3, 148.04) = 30.57, *p* < 0.001. For a more comprehensible depiction of the experimental effects, the adjusted mean scores for the three experimental groups after eliminating the effect of the covariates were determined (see Figure 6). After controlling for the covariates, the experimental group that had learned with static representations had an adjusted mean posttest score of *M*_*adj*_ = 6.12, while the groups learning with linear dynamic and interactive dynamic visualizations had respective adjusted mean posttest scores of *M_*adj*_* = 7.20 and *M*_*adj*_ = 7.34.

**TABLE 3:** Hierarchical regression supplemented by contrasts.

	Posttest score	
Predictor	*ΔR^2^*	ß
**Step 1**	0.381***	
Graph comprehension		0.27***
Intelligence		0.37***
Intrinsic motivation to learn mathematics		0.28***
**Step 2**	0.032*	
Contrast 1 (S vs. LD/ID)		0.18**
Contrast 2 (LD vs. ID)		0.02
Total *R*^2^	0.413***	
*N*	152	

S = static; LD = linear dynamic; ID = interactive dynamic. The coefficients of determination and the regression coefficients are pooled values based on five imputations. * p < 0.05, ** p < 0.01, *** p < 0.001.

In order to determine whether the adjusted mean posttest scores differed significantly between the groups, orthogonal contrasts were inserted into the second regression analysis. Since the number of subjects in the experimental groups was not completely balanced (static: *n*_1_ = 54, linear dynamic: *n*_2_ = 50, interactive dynamic: *n*_3_ = 48), the contrasts were adjusted to the size of the experimental groups. Therefore, for the comparison of static representations and dynamic (linear dynamic or interactive dynamic) visualizations, the contrast coefficient K_1_ = (-98, 54,54) was used, whereas the linear dynamic and the interactive dynamic group were compared using the contrast coefficient K_2_ = (0, -48, 50). Thus, the sum of the weighted contrast products was 0, and the tested hypotheses were non-redundant and independent (c.f., Pedhazur, 1997).

The integration of orthogonal contrasts contributed significantly to a 3.2 percentage points increase in explained variance of the posttest score, *F*(2, 149.04) = 4.00, *p* = 0.02. This means that experimental group had a significant effect on posttest scores. Specifically, there was a significant difference in learning between static and dynamic visualizations, β = 0.18, *t*(145.03) = 2.81, *p* = 0.006. However, no significant difference could be identified between learning with linear dynamic and learning with interactive dynamic visualizations, β = 0.02, *t*(145.03) = 0.30, *p* = 0.77.

To verify the robustness of the results, a simple regression analysis was performed in addition to the described covariance analysis. No covariates were included as predictors in this regression analysis. The integration of the contrasts resulted in a significant proportion of the variance explained, at 5.3 percent, *F*(2, 150) = 4.19, *p* = 0.02. Consistent with the covariance analysis, the experimental groups with linear dynamic or interactive dynamic visualizations learned significantly more than the experimental group with static representations did, β = 0.21, *t*(150) = 2.70, *p* = 0.008, whereas there was no significant difference in learning between linear dynamic and interactive dynamic visualizations, β = 0.08, *t*(150) = 1.04, *p* = 0.30.

### Aptitude–Treatment Interactions (H3)

Hypothesis 3 postulated aptitude–treatment interactions between visual-spatial ability and learning with dynamic visualizations. Therefore, a moderated regression analysis (see Table 4) was performed to determine whether visual-spatial ability had a moderator effect. In the first step, the predictors graph comprehension, visual-spatial ability, and intrinsic motivation to learn mathematics, as well as the two orthogonal contrasts, were included in the regression model. These five predictors accounted for 40.4 percent of the variance of the posttest, *F*(5, 146.04) = 19.93, *p* < 0.001; visual-spatial ability showed a significant main effect, β = 0.31, *t*(145.04) = 3.45, *p* < 0.001. In the second step, interactions between the contrasts and visual-spatial ability were included in the regression model. The interaction terms did not significantly contribute to the explained variance, *F*(2, 149.04) = 1.86, *p* = 0.16.

**TABLE 4:** Hierarchical regression for analyzing aptitude–treatment interaction.

	Posttest score	
Predictor	*ΔR^2^*	ß
**Step 1**	0.404***	
Graph comprehension		0.33***
Visual-spatial ability		0.31***
Intrinsic motivation to learn mathematics		0.24***
Contrast 1 (S vs. LD/ID)		0.18**
Contrast 2 (LD vs. ID)		0.02
**Step 2**	0.015	
Contrast 1 × Visual-spatial ability		0.09
Contrast 2 × Visual-spatial ability		–0.13
Total *R*^2^	0.419***	
*N*	152	

S = static; LD = linear dynamic; ID = interactive dynamic. The coefficients of determination and the regression coefficients are pooled values based on five imputations. ** p < 0.01, *** p < 0.001.

## Discussion

### Learning With Dynamic Visualizations

In our experiment, dynamic visualizations were significantly more beneficial for learning than were static representations. Thus, in accordance with Hypothesis 1, an empirically verifiable added value of dynamic visualizations was found. Potential reasons for the effect can be inferred from the design of the learning environment and the dynamic visualizations.

For example, the dynamic visualizations may have functioned as scaffolding for the construction of a satisfactory mental model. The content in the experiment required a relatively high cognitive effort to mentally simulate the dynamic without external support. In the static representation condition, movement of the point along the perimeter of a triangle or quadrilateral had to be simulated and the effects of this variation analyzed and assessed in working memory. An incorrect mental simulation of the dynamic process most likely led to inadequate inferences about the graph’s shape. This result complies with the idea of supplantation (Salomon, 1979/1994) that was assumed by Vogel et al. (2007) und Hoffkamp (2011) as a theoretical underpinning of dyna-linking. Dynamic visualizations are conducive to learning if they supplant a mental process the student is unable to perform. Therefore, dynamic visualizations can be used to overcome a hurdle in learning mathematics. Conversely, dynamic visualizations do not show a positive learning effect if students do not need supplantation, that is, that they can carry out the necessary mental processes successfully without a dynamic visualization.

Furthermore, the content in the learning environment was developed gradually in all three experimental groups. The students in each group first had to anticipate the form of the graph. The correct graph became visible in a subsequent task. The group learning with static representations could hence also see whether their mental simulation of the dynamic process was correct. In contrast with the experimental groups learning with dynamic visualizations, however, the static representations group had very little opportunity to understand why their considerations may have been wrong; those in the dynamic visualizations groups could contemplate the dynamic on the screen, subsequently correct any erroneous considerations and ideally explore explanations for the shape of the graph. In the dynamic visualization of the equilateral triangle, for example, students could observe that in the middle section the length of the chord decreased more and more slowly until a local minimum was reached; and that the length of the chord then increased speed until it reached a local maximum in the next corner. Being able to observe this process in the dynamic visualization groups made it easier for these learners to realize that the graph in the middle section had to have a symmetrical convex shape with a local minimum in the middle. The group learning with static representations, on the other hand, could only observe that the graph had a convex symmetric form with a local minimum in the following task. If these learners did not correctly anticipate this form (e.g., due to faulty mental simulation of the dynamic process), no help was available to generate a satisfactory mental model and to understand why the graph shape presented was correct. To draw conclusions solely from the illustrated form of the graph about why their mental simulation of the dynamic process was faulty would have required a considerable, in some cases excessive, amount of cognitive effort from the learners. Therefore, dynamic visualizations may have enabled the other student groups to construct a more meaningful and coherent model of the learning content.

Hypothesis 2 could not be corroborated as no difference between learning with interactive and learning with linear dynamic visualizations was found. A greater learning effect of interactive dynamic visualizations was postulated primarily for two reasons. First, it was assumed that interactive dynamic visualizations would make it possible to control and investigate the aspects that were relevant to the particular problem more precisely (Ploetzner and Lowe, 2004) and therefore induce a deeper processing of the learning content (Palmiter and Elkerton, 1993). When asked about the location of the local minima of the length of the chord, for example, the chord could be manipulated more precisely and repeatedly at the relevant point. When using a linear dynamic visualization, the visualization had to be observed carefully; the transitory moment at which the chord became minimal could not be missed. Overall, it seems that the transitivity of the linear dynamic visualization (Höffler and Leutner, 2011) had no negative effect on learners. It seemed that the learners did not experience additional difficulties in processing the changes in the linear dynamic visualization, as shown in some previous research (cp. Bétrancourt and Tversky, 2000). In our experiment, it was just as beneficial to observe the dynamic process in a linear dynamic visualization as it was to work with an interactive dynamic visualization.

Despite this, the experiment also showed that interactivity had no negative effects. Under the assumption that interactivity ties up cognitive resources unavailable for the learning process (Ploetzner and Lowe, 2004), a negative effect of interactive compared with linear dynamic visualizations would theoretically have been understandable. One reason for the non-negative effect of interactivity could be that the interaction possibilities in the experiment were implemented very sparingly, and thus, the interactivity caused no relevant higher cognitive load. Learners could only move the point on the perimeter of the triangle. Other interactive design options (e.g., moving the corner points of the figure or shifting the starting point of the chord) were intentionally disabled to keep the cognitive load and potential negative effects caused by the interaction option low.

In sum, the theoretically assumed advantage of interactive dynamic visualizations over linear dynamic visualizations could not be proven empirically. The potential of interactivity might only come to light in more complex and multifaceted tasks like Hoffkamp’s (2011). In these tasks, the learners could be more able to regulate the cognitive load imposed by a dynamic visualization through interactive actions. Furthermore, the possibility to investigate a task more focussed in an interactive dynamic visualization may come more into play with a variety of interaction options because they enable students to focus their attention on a particular feature of the dynamic visualization.

### Aptitude–Treatment Interaction

Regarding Hypothesis 3, no significant aptitude-treatment interaction between visual-spatial ability and learning with dynamic visualizations was found, despite a significant main effect of visual-spatial ability in our experiment. Therefore, a one-directional effect, as assumed by the ability-as-enhancer or the ability-as-compensator thesis, could not be corroborated. However, we should point out that the absence of a significant effect did not prove that there is no aptitude-treatment interaction. The two assumed effects might have balance out, that is, that both an enhancing and a compensating effect of visual-spatial ability on learning with dynamic visualizations exist. Furthermore, the non-significance could be caused by a lack of power of the experiment. It seems unlikely that our findings were the result of the scales of visual-spatial ability used, as in Sanchez and Wiley (2014) experiment, since we selected several subscales that covered various sub-factors (c.f., Carroll, 1993) of visual-spatial ability.

### Limitations

The intervention in the experiment only took 25 min. Hence, it was a relatively short and limited learning process. This raises the question of how sustainable the learning process induced by dynamic visualization really was. On the one hand, the differences in learning gains may add up in longer learning units; that is, that the difference between learning with static representations and learning with dynamic visualization becomes even greater in longer learning units. On the other hand, dynamic visualizations might only enable faster access to the content. In a longer intervention, after a slower “ignition phase,” the group learning with static representations could reach a level as high as that reached by the groups learning with dynamic visualization. One might consider examining which of these two effects occurs during prolonged interventions in a further experiment.

### Research Desiderata

The main intention of the experiment was to find any empirical evidence for the effect of dynamic visualizations vs. static representations in learning essential mathematical content. Despite its success, just a modest effect of dynamic visualizations compared with static representations was found. Many aspects concerning dynamic visualizations in learning and teaching mathematics remain unclear.

First, the conditions under which dynamic visualizations in mathematics education are conducive to learning have not yet been satisfactorily clarified. It has already been suggested that limiting the interaction possibilities appears to prevent excessive cognitive load. A further experiment might elucidate the question of how an excessive level of interaction might hinder learning. Our experiment also did not show that interactive dynamic visualizations are more beneficial than linear dynamic visualizations. Experimental studies that take a closer look at comparisons between interactive dynamic and linear dynamic visualizations are therefore desirable.

Furthermore, the experiment was based on the assumption that a didactically designed learning environment is needed to generate positive learning effects of dynamic visualizations. Therefore, the dynamic visualizations were integrated into a learning environment in which the students had to explore tasks with increasing difficulty and complexity. This approach could also be validated or falsified by means of further empirical investigation. Two experimental groups could work with the same interactive dynamic visualization: one could work freely and without concrete content-related problems with an interactive dynamic visualization (possible task: “Explore the computer-based learning environment and describe what discoveries you make”); while the other could be given pre-structured and targeted assignments. Such a design could be used to determine to which extent simply exploring an interactive dynamic visualization itself induces a learning process.

Finally, it would be beneficial to investigate the learning effect of dynamic visualizations for further mathematical content. These studies should be combined with further in-depth theoretical considerations about the advantages that learning with dynamic visualizations can offer regarding these contents. For example, in calculus, many students struggle to comprehend limiting processes (e.g., derivative, integral). Therefore, several dynamic visualizations are available to support the learning and teaching of calculus. Against the backdrop of our quantitative results and findings based on qualitative research from Hoffkamp (2011), it seems plausible to assume that the appropriate use of dynamic visualizations could be beneficial in teaching calculus. However, an empirical validation with quantitative experiments of the effectiveness of teaching and learning with dynamic visualizations in calculus is still pending. Furthermore, the use of dynamic visualizations for learning dynamic aspects in stochastics (e.g., the law of large numbers or central limit theorem) or geometry (e.g., construction tasks) has not yet been sufficiently empirically investigated.

## Conclusion

Eventually, we can draw some conclusions for teaching mathematics from the present study. On the one hand, we can state that, under certain conditions, dynamic visualizations can support learning better than static representations. For example, embedding dynamic visualizations into an elaborated learning environment seems beneficial. In consequence, through the interactive relationship between dynamic visualization as an artifact and the tasks, the dynamic visualization can transform into an instrument that enables learning (Rabardel, 2002). It is reasonable to assume that other mathematical content (e.g., calculus, probability theory) can bring out this potential of dynamic visualizations as well.

On the other hand, the effect of dynamic visualizations was rather modest, and interactivity had no additional effect at all. Other cognitively activating features in a learning environment like predict-observe-explain (Urban-Woldron, 2014) could have a higher effect on learning mathematics than dynamic visualizations. Therefore, the present study confirms that expectations in using dynamic visualizations in teaching mathematics should be realistic: Dynamic visualizations are no magic bullets, but to a certain degree, they can facilitate learning processes in mathematics.

## Author’s Note

This manuscript is based on dissertation research (Rolfes, 2018) directed by the second and third authors of the manuscript.

## Data Availability Statement

The datasets generated for this study are available on request to the corresponding author.

## Ethics Statement

This study was carried out in accordance with the guidelines for scientific studies in schools in the German state Rhineland-Palatinate (*Wissenschaftliche Untersuchungen an Schulen in Rheinland-Pfalz*), Aufsichts- und Dienstleistungsdirektion Trier. The protocol was approved by the Aufsichts- und Dienstleistungsdirektion Trier. All subjects have provided written informed consent in accordance with the Declaration of Helsinki.

## Author Contributions

All authors listed have made a substantial, direct and intellectual contribution to the work, and approved it for publication.

## Conflict of Interest

The authors declare that the research was conducted in the absence of any commercial or financial relationships that could be construed as a potential conflict of interest.
