# Supplementary material for: Learning the Concept of Function With Dynamic Visualizations
Source: Front Psychol. 2020 Apr 30;11:693. doi: 10.3389/fpsyg.2020.00693 (PMC7212367; doi:10.3389/fpsyg.2020.00693)
Supplement: Supplementary file 1 [file Data_Sheet_1.PDF]

Supplementary Material 1: Learning Environment

The intervention of the experiment was designed as an HTML-based self-learning environment. There were three experimental variations of the learning environment. An interactive GeoGebra applet was loaded into the left frame of the HTML page in the interactive dynamic visualization group. The point Q could be moved with the mouse. In the linear dynamic visualization group, a play button was displayed below the applet, in which point Q ran once through the edge line of the triangle with constant speed. The linear dynamic visualization could be started repeatedly. The same illustration was used in the third experimental group with the static representation, but point Q was fixed and could not be moved. The textual parts that varied between the experimental groups are highlighted yellow in the following screenshots.

Screenshot of task 1 in the interactive dynamic visualization group

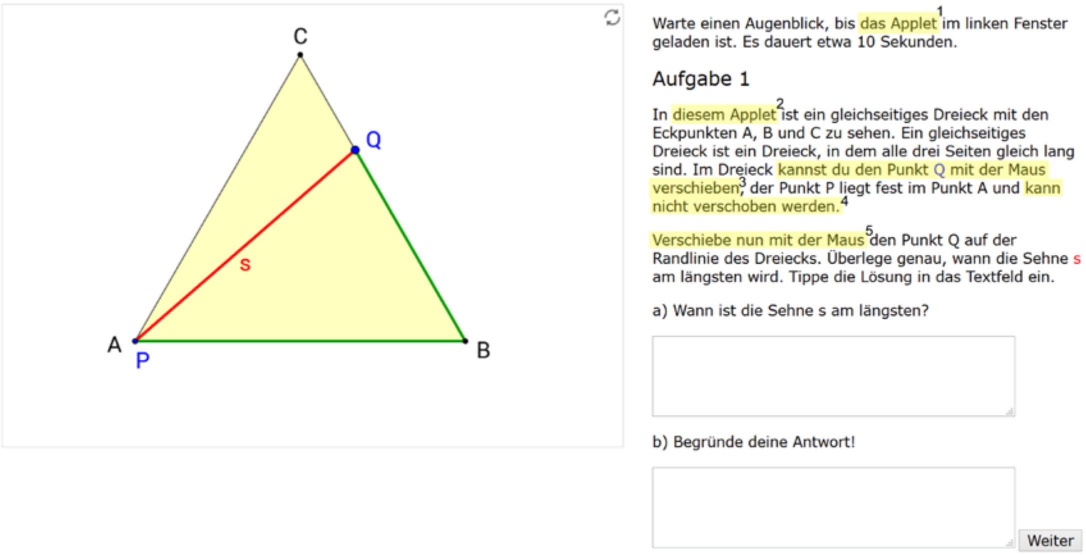

Textual variation in the linear dynamic visualization group:
[1]: die Animation
[2]: dieser Animation
[3]: verschiebt sich der Punkt Q in der Animation
[4]: wird nicht verschoben
[5]: Animiere nun durch Klick auf den Play-Knopf

Textual variation in the static representation group:
[1]: die Grafik
[2]: dieser Grafik
[3]: soll der Punkt Q in Gedanken verschoben werden
[4]: soll nicht verschoben werden können
[5]: Verschiebe in Gedanken

Screenshot of task 2 in the interactive dynamic visualization group

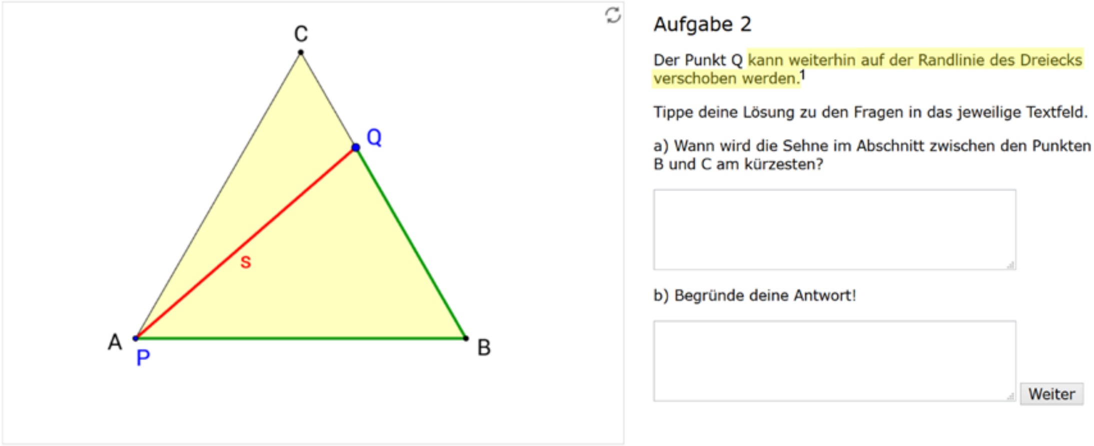

Textual variation in the linear dynamic visualization group:

[1]: per Klick auf Play animiert werden

Textual variation in the static representation group:

[1]: in Gedanken auf der Randlinie des Dreiecks verschoben werden

Screenshot of task 3 in the interactive dynamic visualization group

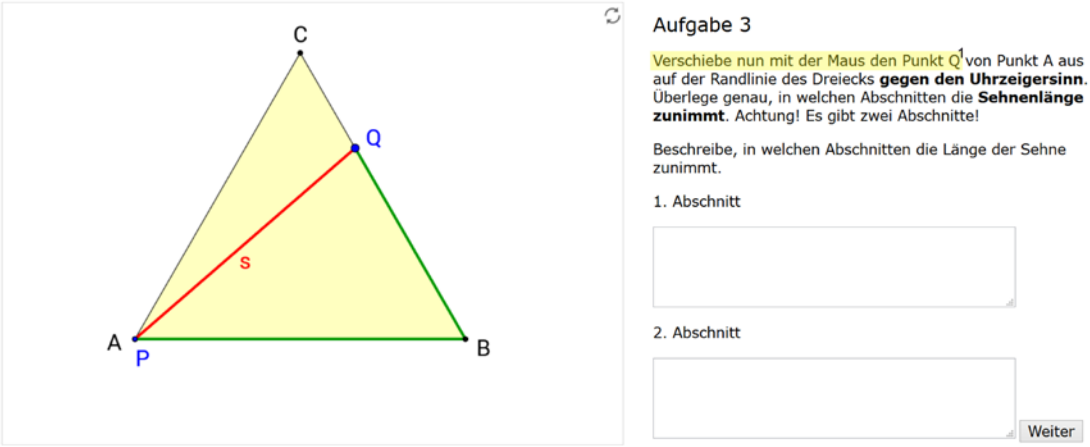

Textual variation in the linear dynamic visualization group:

[1]: In der Animation bewegt sich der Punkt Q

Textual variation in the static representation group:

[1]: Verschiebe nun in Gedanken den Punkt Q

Screenshot of task 4 in the interactive dynamic visualization group

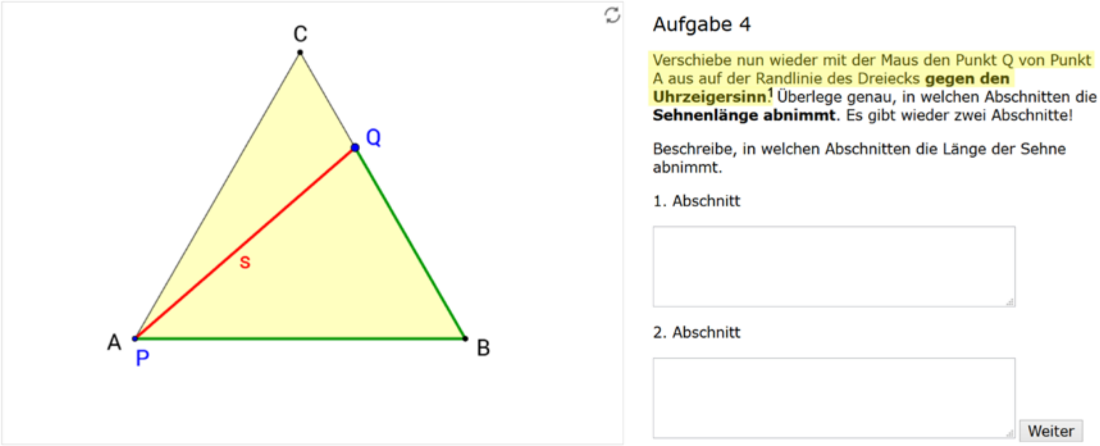

Textual variation in the linear dynamic visualization group:
[1]: Animiere nur wieder mit Klick auf den Play-Button Punkt Q, damit er sich von Punkt A aus auf der Randlinie des Dreiecks **gegen den Uhrzeigersinn** bewegt.

Textual variation in the static representation group:
[1]: Verschiebe nun wieder in Gedanken den Punkt Q von Punkt A aus auf der Randlinie des Dreiecks **gegen den Uhrzeigersinn**.

Screenshot of task 5 in the interactive dynamic visualization group

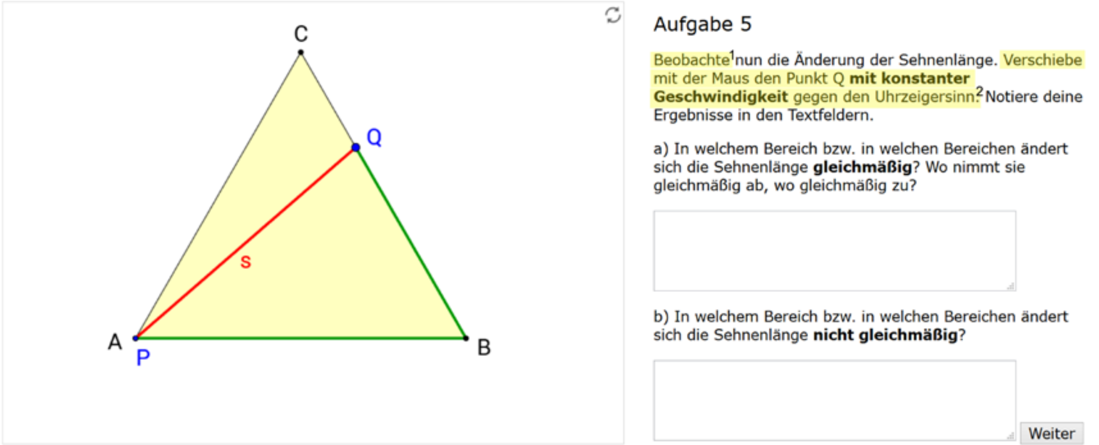

Textual variation in the linear dynamic visualization group:
[1]: Klicke auf den Play-Knopf, damit sich der Punkt Q **mit konstanter Geschwindigkeit** gegen den Uhrzeigersinn bewegt.

Textual variation in the static representation group:
[1]: Verschiebe in Gedanken den Punkt Q **mit konstanter Geschwindigkeit** gegen den Uhrzeigersinn.

Screenshot of task 6 in the interactive dynamic visualization group

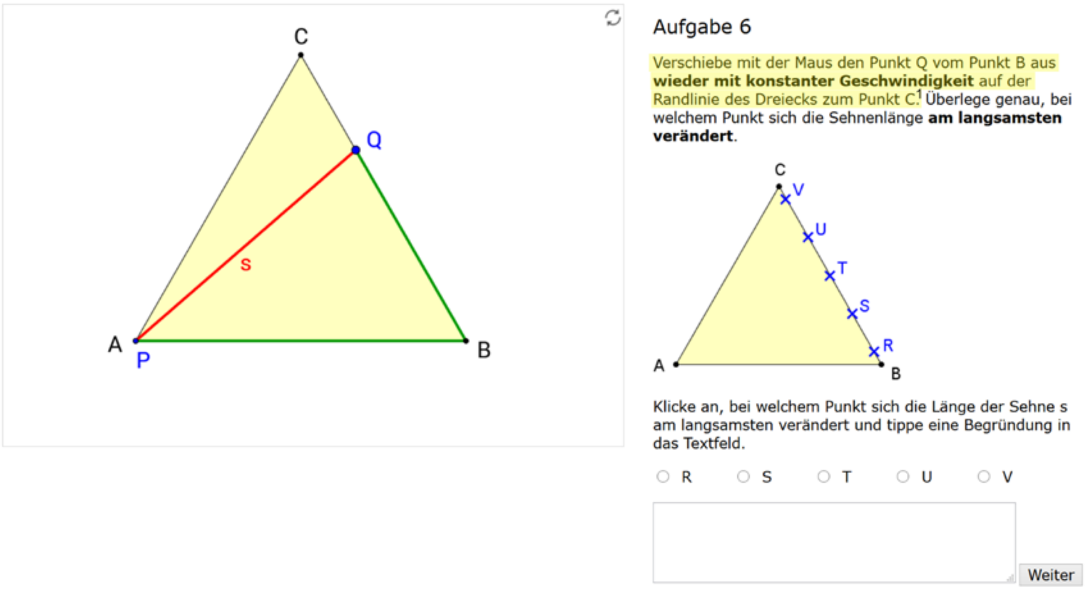

Textual variation in the linear dynamic visualization group:
[1]: Klicke wieder auf den Play-Button, damit sich der Punkt Q vom Punkt B aus **wieder mit konstanter Geschwindigkeit** auf der Randlinie des Dreiecks zum Punkt C bewegt.

Textual variation in the static representation group:
[1]: Verschiebe in Gedanken den Punkt Q vom Punkt B aus **wieder mit konstanter Geschwindigkeit** auf der Randlinie des Dreiecks zum Punkt C.

Screenshot of task 7 in the interactive dynamic visualization group

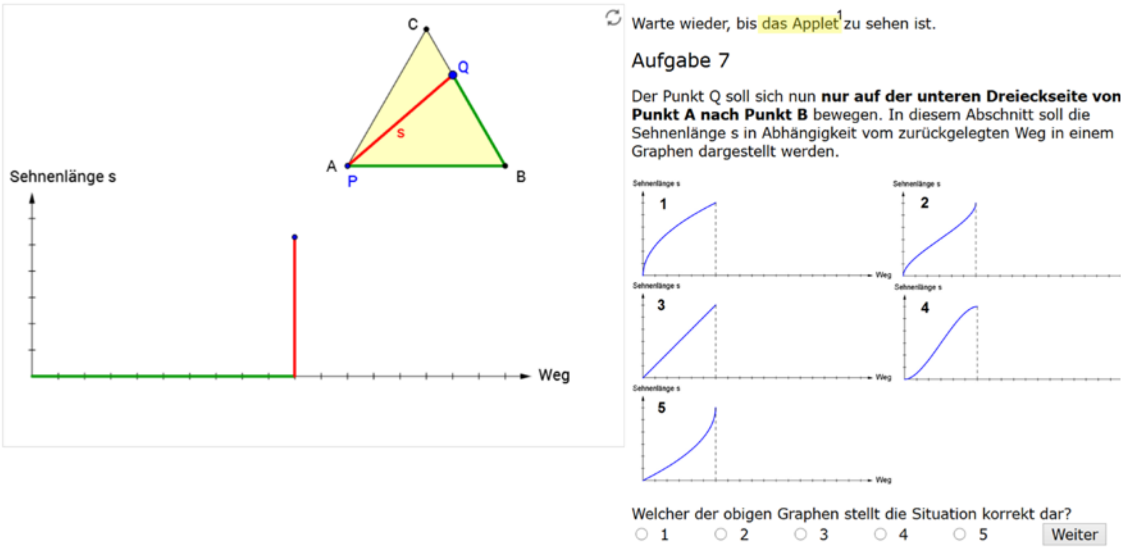

Textual variation in the linear dynamic visualization group:
[1]: die Animation

Textual variation in the static representation group:
[1]: die Grafik

Screenshot of task 8 in the interactive dynamic visualization group

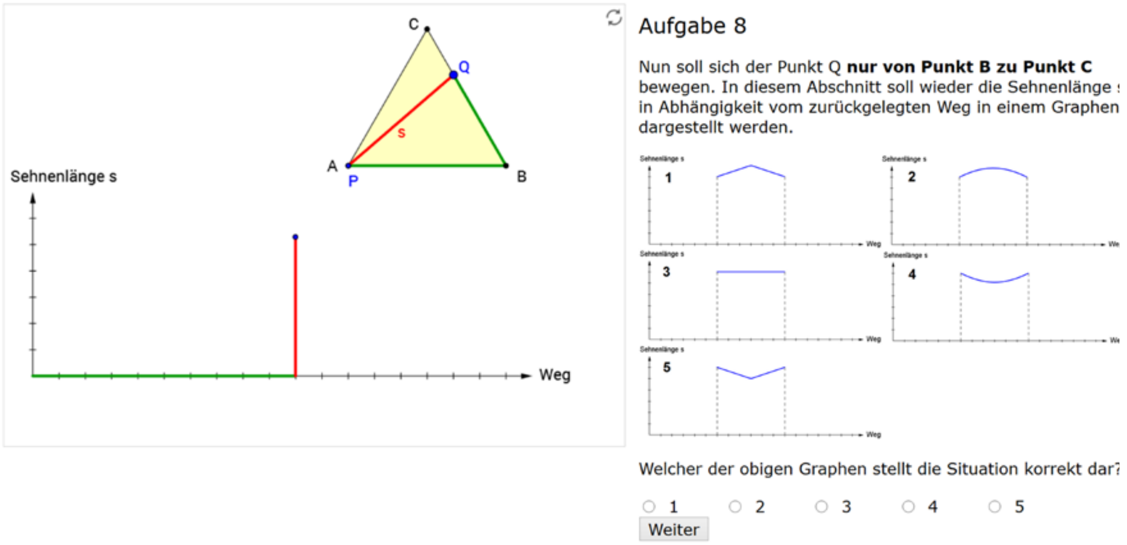

No textual variation in the linear dynamic visualization group and the static representation group

Screenshot of task 9 in the interactive dynamic visualization group

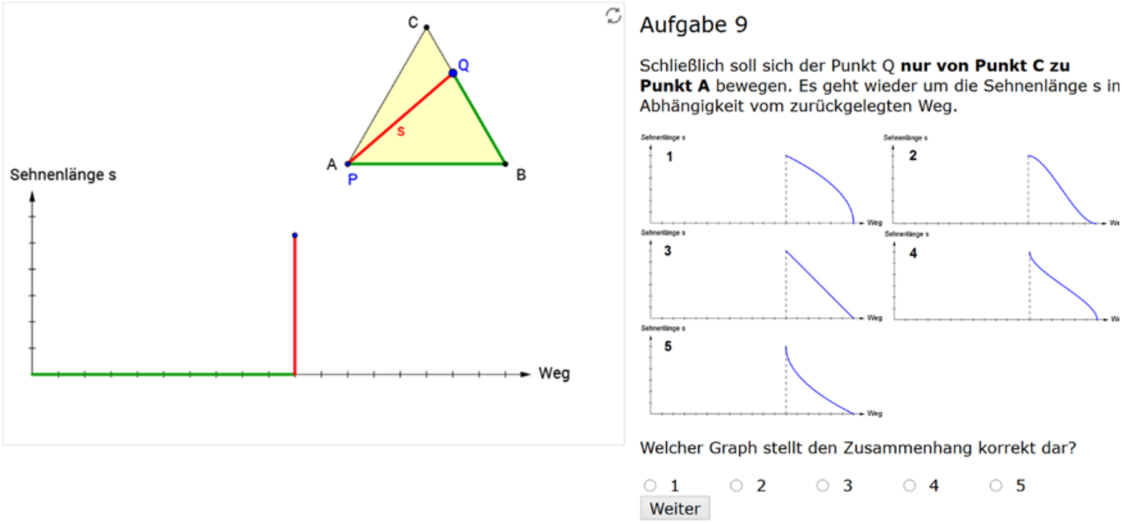

No textual variation in the linear dynamic visualization group and the static representation group

Screenshot of task 10 in the interactive dynamic visualization group

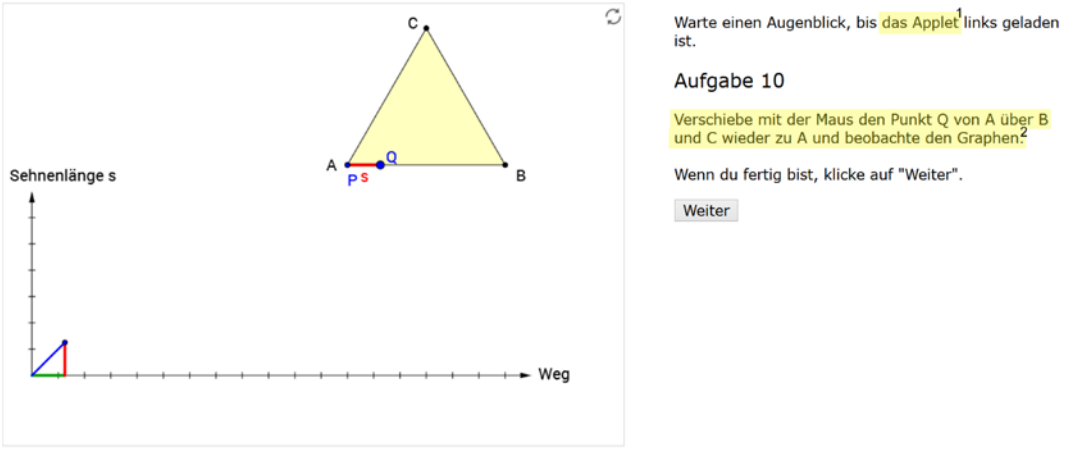

Textual variation in the linear dynamic visualization group:

[1]: die Animation

[2]: Starte die Animation von Punkt Q, der von A über B und C wieder zu A wandert, und beobachte den Graphen.

Textual variation in the static representation group:

[1]: die Grafik

[2]: Verschiebe in Gedanken den Punkt Q von A über B und C wieder zu A und stelle dir den Graphen vor.

Screenshot of task 11 in the interactive dynamic visualization group

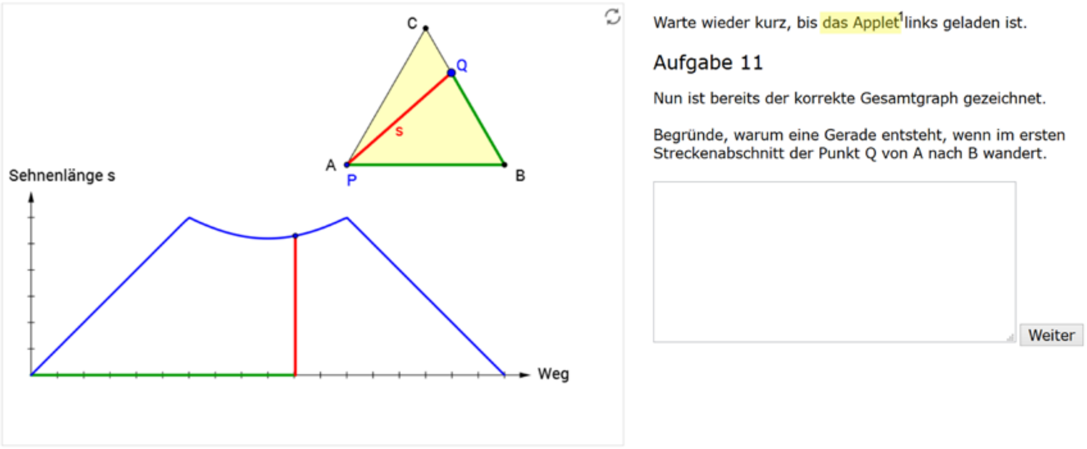

Textual variation in the linear dynamic visualization group:

[1]: die Animation

Textual variation in the static representation group:

[1]: die Grafik

Screenshot of task 12 in the interactive dynamic visualization group

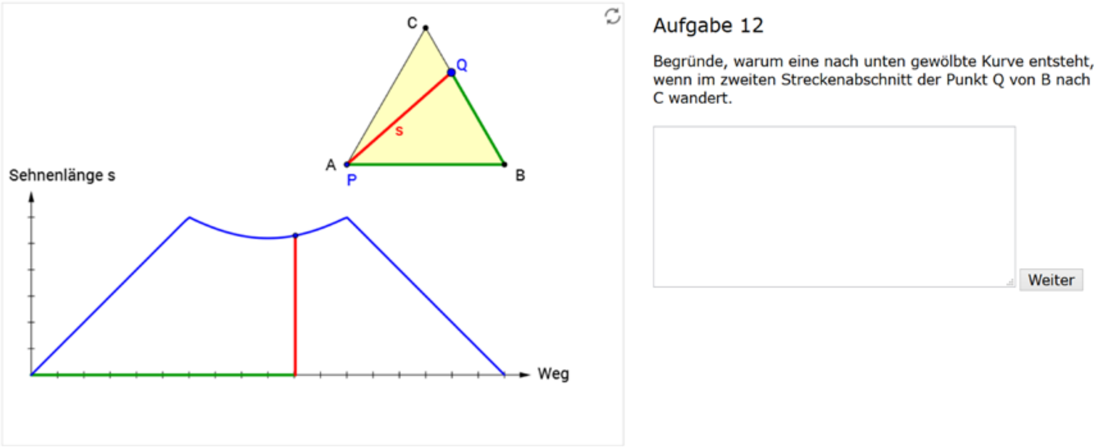

No textual variation in the linear dynamic visualization group and the static representation group

Screenshot of task 13 in the interactive dynamic visualization group

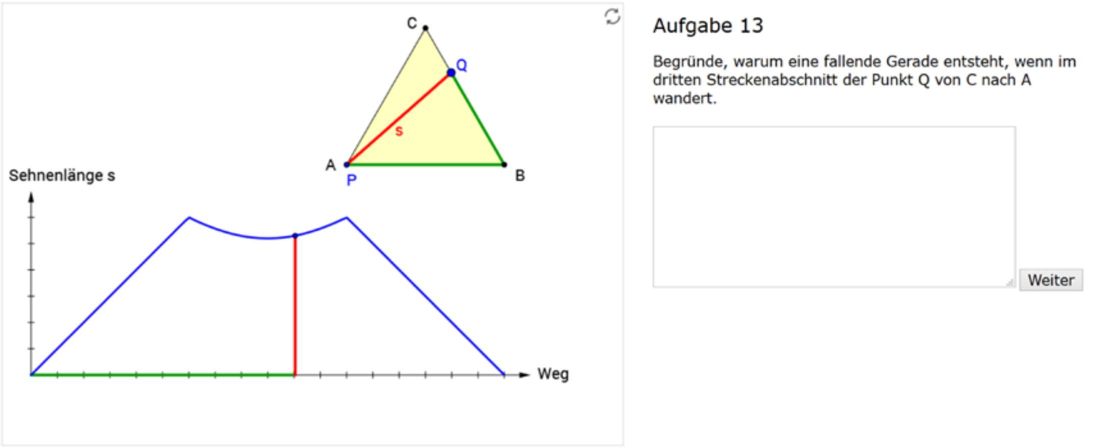

No textual variation in the linear dynamic visualization group and the static representation group

Screenshot of task 14 in the interactive dynamic visualization group

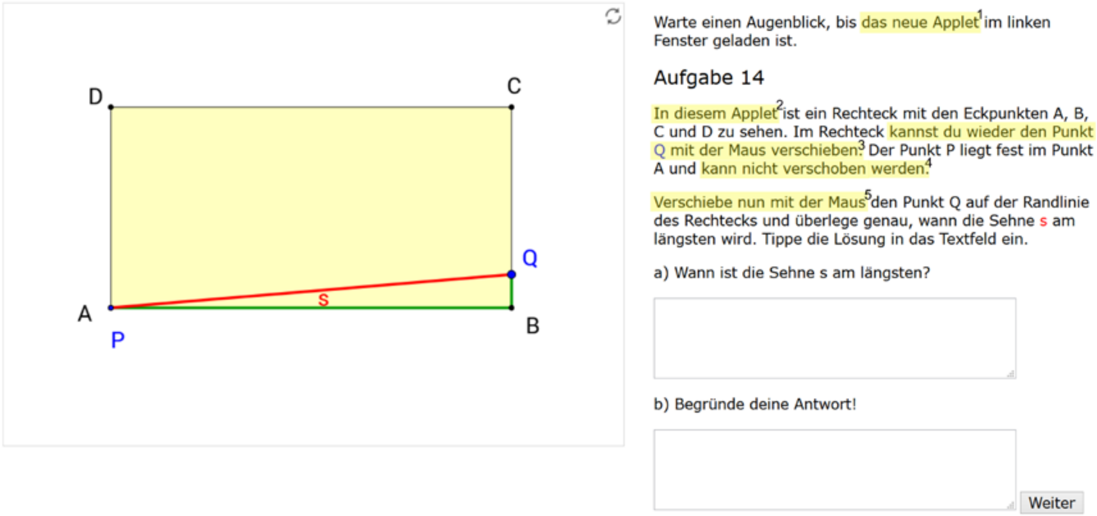

Textual variation in the linear dynamic visualization group:

[1]: die neue Animation

[2]: In dieser Animation

[3]: wird wieder der Punkt Q durch Klick auf den Play-Knopf verschoben

[4]: wird nicht verschoben

[5]: Animiere nun durch Klick auf den Play-Knopf

Textual variation in the static representation group:

[1]: die neue Grafik

[2]: In dieser Grafik

[3]: sollst du wieder den Punkt Q in Gedanken verschieben

[4]: soll nicht verschoben werden

[5]: Verschiebe nun in Gedanken

Screenshot of task 15 in the interactive dynamic visualization group

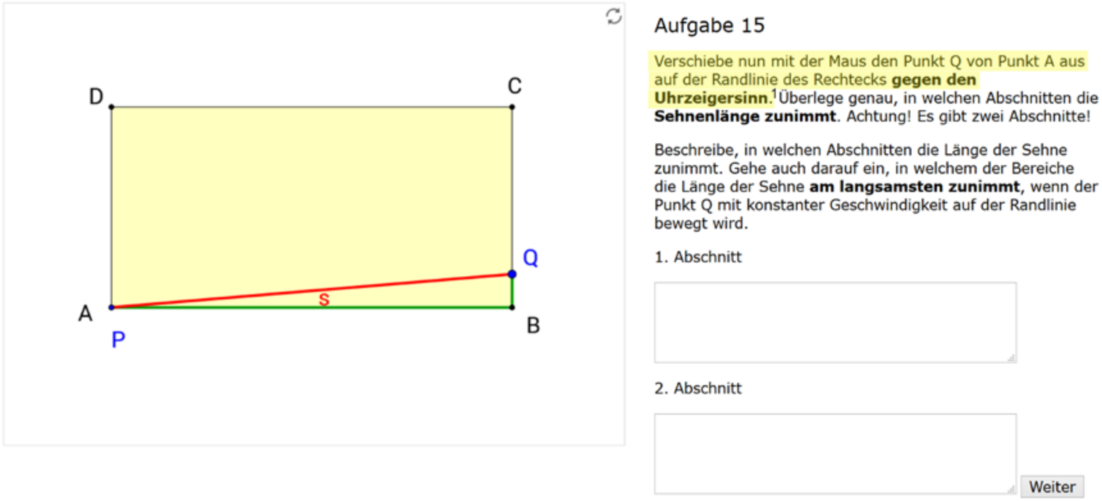

Textual variation in the linear dynamic visualization group:
[1]: Klicke auf den Play-Knopf, damit sich der Punkt Q von Punkt A aus auf der Randlinie des Rechtecks **gegen den Uhrzeigersinn** verschiebt.

Textual variation in the static representation group:
[1]: Verschiebe nun in Gedanken den Punkt Q von Punkt A aus auf der Randlinie des Rechtecks **gegen den Uhrzeigersinn**.

Screenshot of task 16 in the interactive dynamic visualization group

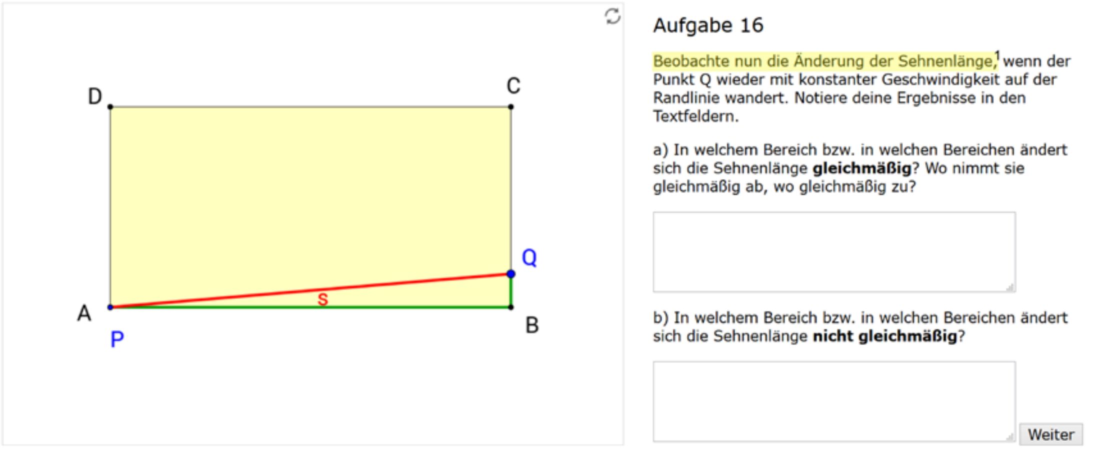

Textual variation in the linear dynamic visualization group:
[1]: Beobachte nun die Änderung der Sehnenlänge

Textual variation in the static representation group:
[1]: Nun geht es um die Änderung der Sehnenlänge

Screenshot of task 17 in the interactive dynamic visualization group

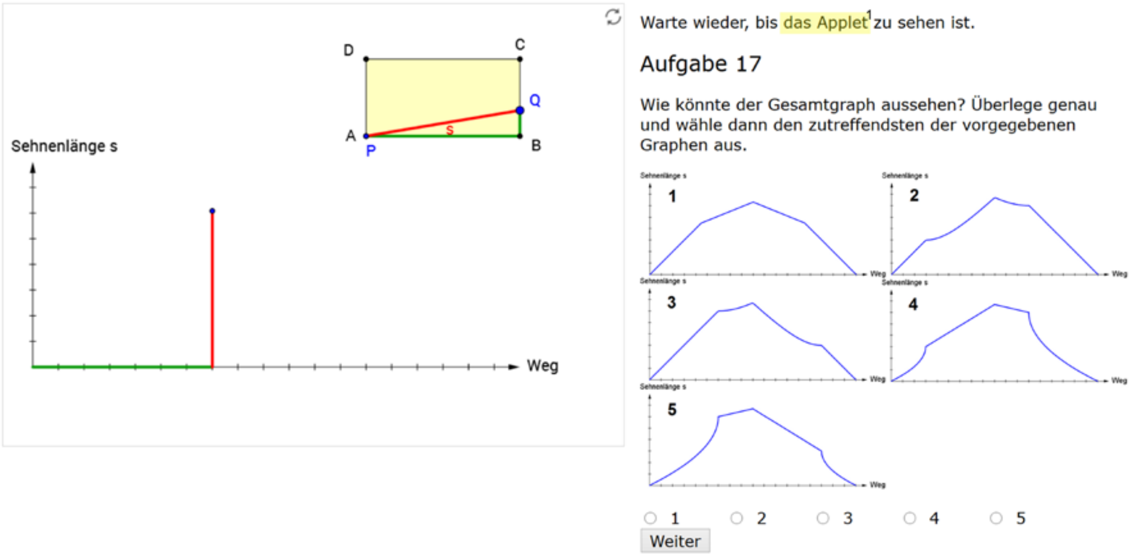

Textual variation in the linear dynamic visualization group:

[1]: die Animation

Textual variation in the static representation group:

[1]: die Grafik

Screenshot of task 18 in the interactive dynamic visualization group

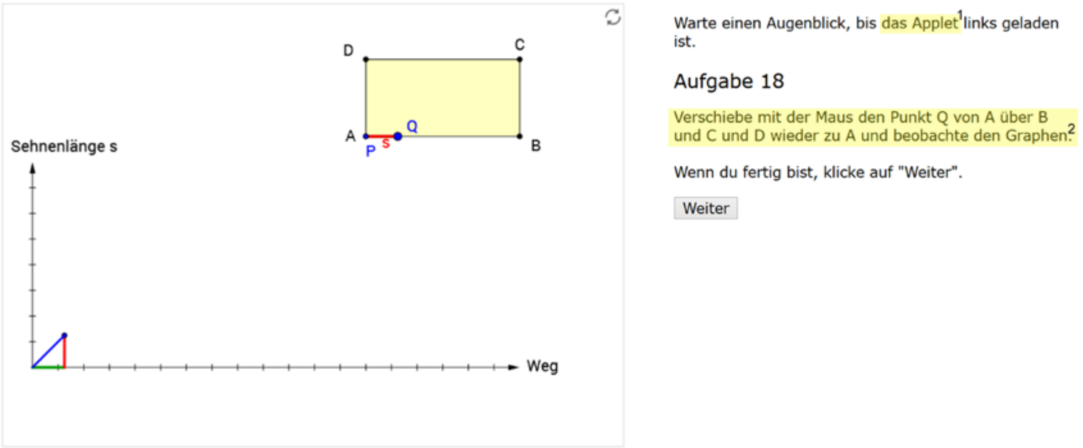

Textual variation in the linear dynamic visualization group:

[1]: die Animation

[2]: Animiere durch Klick auf den Play-Knopf den Punkt Q, damit er sich von Punkt A über B und C und D wieder zu A verschiebt und beobachte den Graphen.

Textual variation in the static representation group:

[1]: die Grafik

[2]: Verschiebe in Gedanken den Punkt Q von A über B und C und D wieder zu A und stelle dir den Graphen vor.

Screenshot of task 19 in the interactive dynamic visualization group

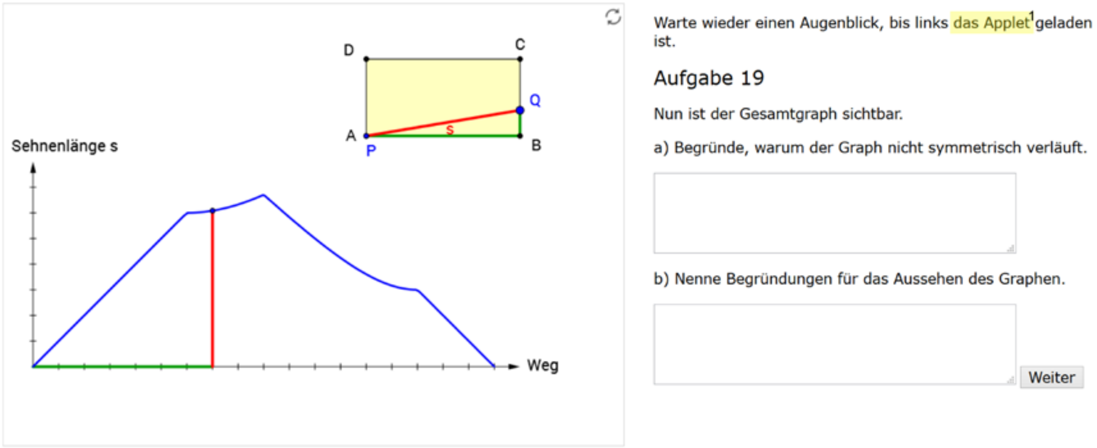

Textual variation in the linear dynamic visualization group:
[1]: die Animation

Textual variation in the static representation group:
[1]: die Grafik

(Source: Rolfes, T. (2018). *Funktionales Denken: Empirische Ergebnisse zum Einfluss von statischen und dynamischen Repräsentationen. Anhang.* Landau: Autor. https://doi.org/10.13140/RG.2.2.26605.64485)
