# Supplementary material for: Learning the Concept of Function With Dynamic Visualizations
Source: Front Psychol. 2020 Apr 30;11:693. doi: 10.3389/fpsyg.2020.00693 (PMC7212367; doi:10.3389/fpsyg.2020.00693)
Supplement: Supplementary file 2 [file Data_Sheet_2.PDF]

Supplementary Material 2: Graph Comprehension Test

Aquarium

Martina füllt ein rechteckiges Aquarium mithilfe von zwei Schläuchen. Die Schläuche füllen das Aquarium mit der selben Füllgeschwindigkeit. Als das Aquarium halbvoll ist, dreht sie einen Schlauch zu, ändert aber die Füllgeschwindigkeit des anderen Schlauchs nicht.

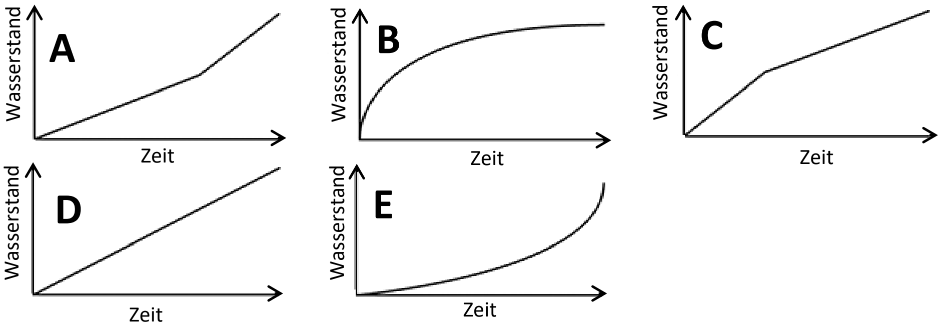

Welcher der oben abgebildeten Graphen stellt am besten dar, wie sich der Wasserstand über die Zeit verändert?

☐ A

☐ B

☐ C

☐ D

☐ E

Quelle des Items: deutsche Übersetzung des Items mit der Question-ID 2013-8M3#8M150501 von U.S. Department of Education, Institute of Education Sciences, National Center for Education Statistics. (2013). *National Assessment of Educational Progress (NAEP) - 2013 Mathematics Assessment.* Washington, DC: Autor. Verfügbar unter https://nces.ed.gov/NationsReportCard/nqt/Search

Kerze

Nebenstehend ist eine pyramidenförmigen Kerze abgebildet Die Kerze wird angezündet. Zeichne in das untenstehende Koordinatensystem den Graphen für die Höhe der Kerze im Verlauf der Zeit ein.

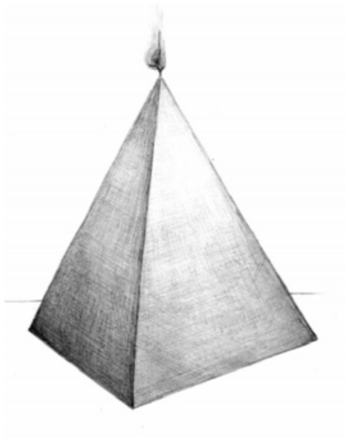

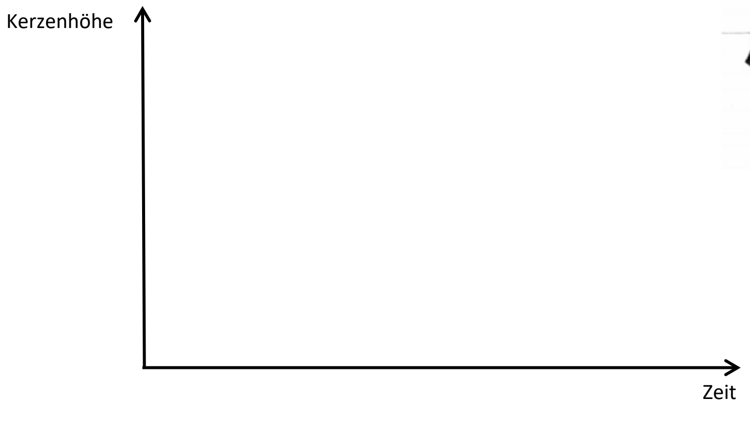

Quelle des Items: Eigenentwicklung

Zeitungen verkaufen

In Zedland gibt es zwei Zeitungen, die Verkäufer einstellen. Die nebenstehenden Anzeigen zeigen, wie die Verkäufer bezahlt werden.

ZEDLAND ALLGEMEINE ZEITUNG (ZAZ)	ZEDLAND NACHRICHTEN
Brauchen Sie Geld? Verkaufen Sie unsere Zeitung! So werden Sie bezahlt: 0,20 Zeds für die ersten 240 Zeitungen, die Sie in einer Woche verkaufen, plus zusätzlich 0,40 Zeds für jede verkaufte Zeitung darüber hinaus.	**Gut bezahlter Job mit wenig Zeitaufwand!** Verkaufen Sie den Zedland Daily und verdienen Sie 60 Zeds pro Woche, plus zusätzlich 0,05 Zeds pro verkaufter Zeitung.

Jonas möchte sich für eine Stelle als Zeitungsverkäufer bewerben. Er muss sich zwischen Zedland Nachrichten und der Zedland Allgemeine Zeitung (ZAZ) entscheiden.

Welcher der folgenden Graphen stellt am besten dar, wie die beiden Zeitungen ihre Verkäufer bezahlen?

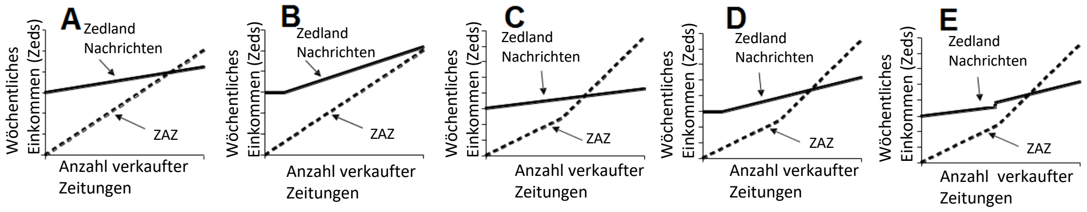

☐ A
☐ B
☐ C
☐ D
☐ E

Quelle des Items: deutsche Übersetzung des Items PM994Q03 der Organisation for Economic Co-operation and Development. (2013). *PISA 2012 released mathematics items*. Paris: Autor. Verfügbar unter http://www.oecd.org/pisa/pisaproducts/pisa2012-2006-rel-items-maths-ENG.pdf

Wassertank

Der Wassertank hat die Form wie auf der Abbildung und hat die angegebenen Maße.
Zunächst ist der Tank leer. Dann wird er mit einer Geschwindigkeit von einem Liter pro Sekunde gefüllt.

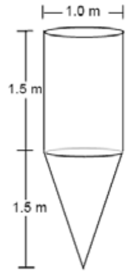

Wassertank

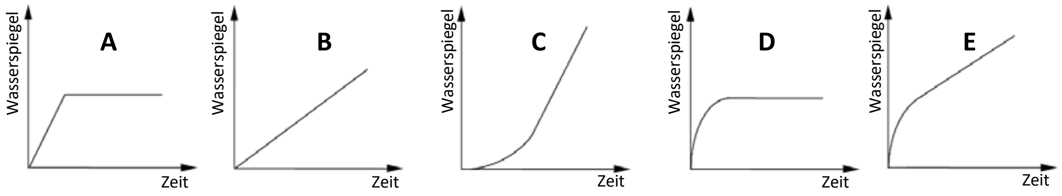

Welcher der oben abgebildeten Graphen zeigt, wie sich der Wasserspiegel mit der Zeit verändert?

☐ A
☐ B
☐ C
☐ D
☐ E

Quelle des Items: deutsche Übersetzung des Items M465 „Water Tank" der Organisation for Economic Co-operation and Development. (2003). *The PISA 2003 Assessment Framework. Mathematics, Reading, Science and Problem Solving Knowledge and Skills.* Paris: OECD Publishing.

Geschwindigkeit

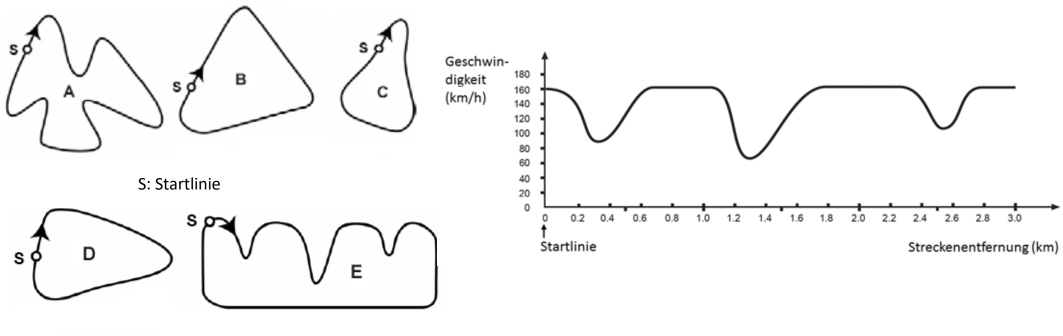

Auf welcher der oben abgebildeten Rennstrecken fuhr der Wagen, sodass der Geschwindigkeitsgraph entstand?

☐ A

☐ B

☐ C

☐ D

☐ E

Quelle des Items: deutsche Übersetzung und leichte Modifikation des Items M159Q05 „Speed of racing car" der Organisation for Economic Co-operation and Development. (2002). *Sample Tasks from the PISA 2000 Assessment: Reading, Mathematical and Scientific Literacy*. Paris: Autor.

Entfernung

Dieser Graph zeigt, wie sich die Geschwindigkeit eines Rennwagens während seiner zweiten Runde auf einer drei Kilometer langen ebenen Rennstrecke verändert.

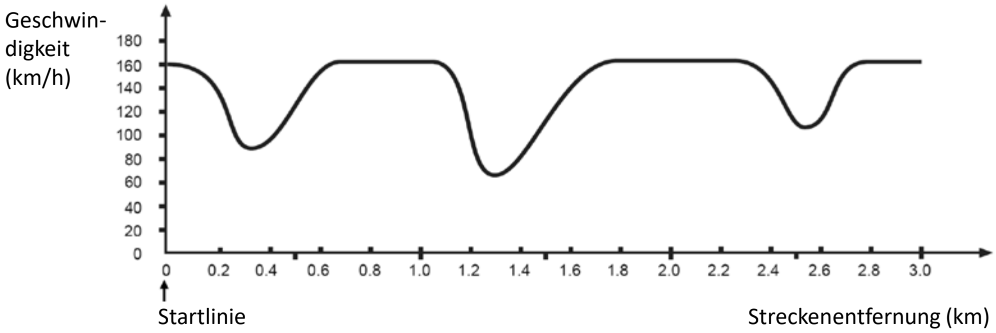

Wie groß ist die ungefähre Entfernung von der Startlinie bis zum Beginn des längsten geradlinigen Abschnitts der Rennstrecke?

☐ 0,5 km

☐ 1,5 km

☐ 2,0 km

☐ 2,3 km

☐ 2,6 km

Quelle des Items: deutsche Übersetzung und leichte Modifikation des Items M159Q01 „Speed of racing car" der Organisation for Economic Co-operation and Development. (2002). *Sample Tasks from the PISA 2000 Assessment: Reading, Mathematical and Scientific Literacy*. Paris: Autor.

Luftlinie

Peter läuft im Kreis um einen Baum.

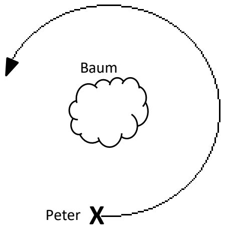

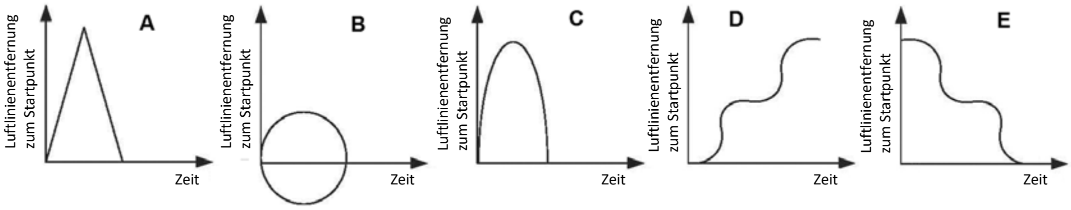

Welcher der Graphen zeigt am besten die Luftlinienentfernung zwischen Peters Startpunkt und seiner Position im Verlauf der Zeit?

☐ A

☐ B

☐ C

☐ D

☐ E

Quelle des Items: Leichte Modifikation des Vera8-Items „Im Kreis laufen“ des Institut für Qualitätsentwicklung im Bildungswesen. (2013). *Beispielaufgaben Mathematik Sek I*. Berlin: Autor. Verfügbar unter https://www.iqb.hu-berlin.de/vera/aufgaben/ma1

Bagger

Für den Keller eines Hauses soll mit zwei Baggern eine Grube ausgehoben werden. Gegen Mittag fällt ein Bagger aus. Die re Arbeit wird noch am gleichen Tag vom verbleibende Bagger geleistet. Skizziere im Koordinatensystem einen Graphen für d ausgehobene Erdmenge in Abhängigkeit von der Zeit.

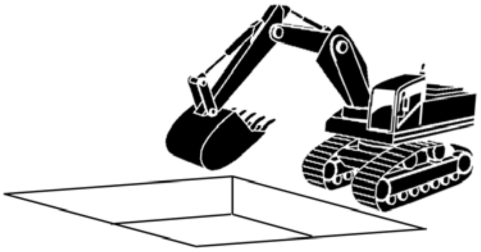

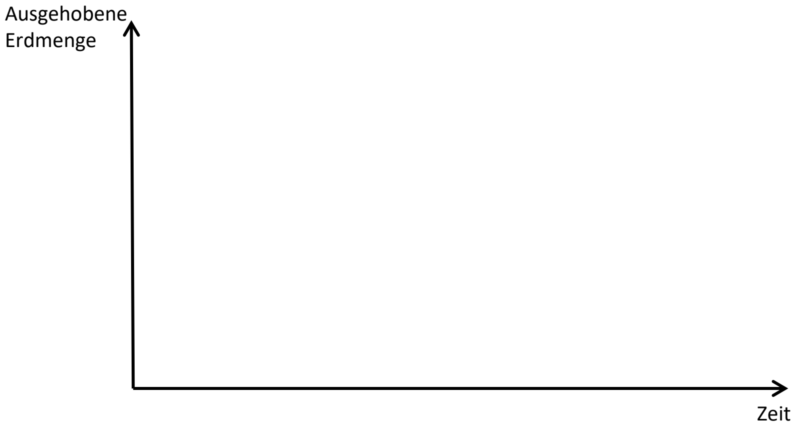

Quelle des Items: Eigenentwicklung

Kurve

Das Auto fährt mit gleichbleibender Geschwindigkeit durch eine Kurve. Der zurückgelegte Weg im Verlauf der Zeit soll in einem Graphen dargestellt werden.

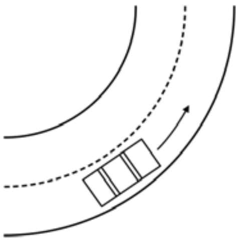

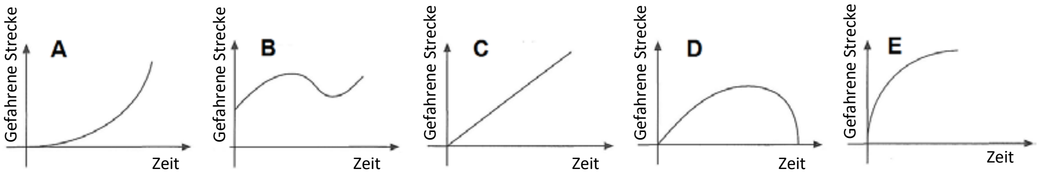

Welcher der oben abgebildeten Graphen stellt die Situation am besten dar?

☐ A
☐ B
☐ C
☐ D
☐ E

Quelle des Items: Leichte Modifikation eines Items aus Schlöglhofer, F. (2000). Vom Foto-Graph zum Funktions-Graph. *Mathematik lehren, 103,* 16–21.

Dreieck

Die gestrichelte Linie wird vom Punkt A aus nach rechts zum Punkt B gezogen. Entsprechend verändert sich der Flächeninhalt der grau markierten Fläche.

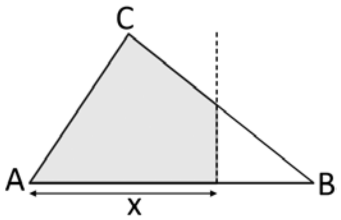

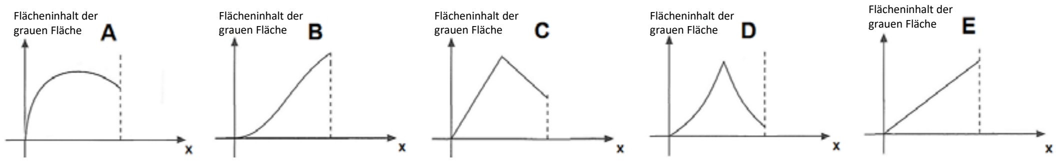

Welcher der oben abgebildeten Funktionsgraphen beschreibt den Zusammenhang am besten?

☐ A
☐ B
☐ C
☐ D
☐ E

Quelle des Items: Leichte Modifikation eines Items aus Schlöglhofer, F. (2000). Vom Foto-Graph zum Funktions-Graph. *Mathematik lehren, 103,* 16–21.

Fähre

Eine Fähre fährt gradlinig mit konstanter Geschwindigkei
der Freiheitsstatue vorbei.

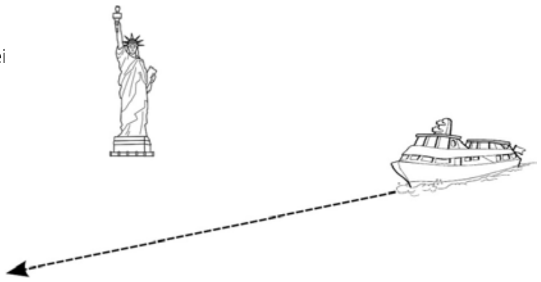

Welcher der folgenden Graphen stellt die Entfernung zwischen Freiheitsstatue und Fähre im Verlauf der Zeit am besten dar?

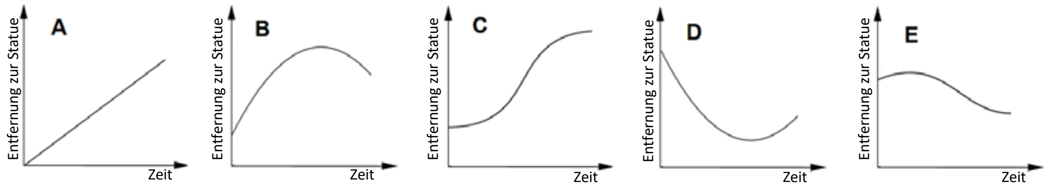

☐ A
☐ B
☐ C
☐ D
☐ E

Quelle des Items: Eigenentwicklung

Angel

Der Angler wirft den Angelhaken
vom Stegrand aus ins Wasser.

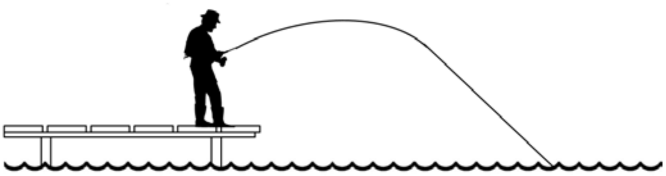

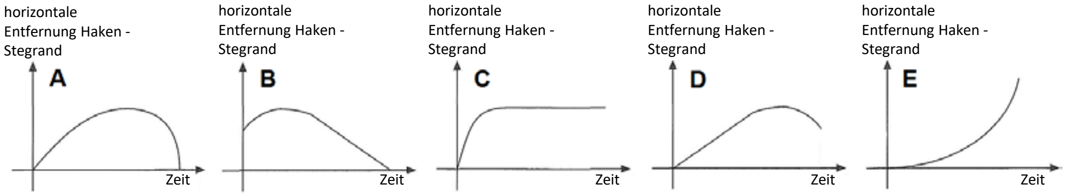

Welcher Graph stellt die horizontale Entfernung vom Angelhaken zum Stegrand beim Auswurf am besten dar?

☐ A
☐ B
☐ C
☐ D
☐ E

Quelle des Items: Leichte Modifikation eines Items aus Schlöglhofer, F. (2000). Vom Foto-Graph zum Funktions-Graph. *Mathematik lehren, 103,* 16–21.

Geschichte zum Graphen

Kreuze die Beschreibung an, die am besten zum Graphen passt.

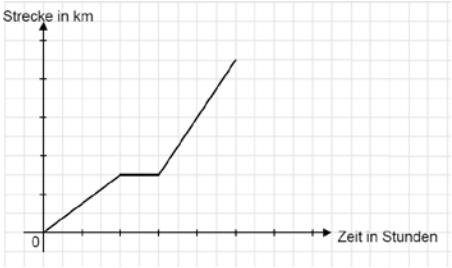

☐ Paula und Sepp machen eine Bergtour. Zuerst steigt der Weg nur wenig an und die beiden kommen gut voran. Dann ist der Weg eine Zeit lang eben. Zum Schluss ist der Weg bis zum Gipfel ziemlich steil und Paula und Sepp kommen nur langsam voran.

☐ Herr Heuer kauft Aktien. Zuerst steigt der Wert der Aktien. Dann bleibt er eine Zeit lang konstant. Schließlich steigt der Wert steil an und Herr Heuer könnte die Aktien mit Gewinn verkaufen.

☐ Lisa und Sven machen eine Radtour. Nach einiger Zeit hat Sven eine Panne und sie müssen sein Rad reparieren. Für den Rest der Strecke fahren beide mit höherer Geschwindigkeit, um die versäumte Zeit aufzuholen.

☐ Lars ist auf dem Weg zur Schule. Unterwegs fällt ihm ein, dass er seinen Taschenrechner vergessen hat, den er für die Mathearbeit braucht. Er läuft zurück nach Hause, nimmt den Taschenrechner und muss sich jetzt beeilen, um pünktlich zur Schule zu kommen.

☐ Familie Schmidt fährt mit dem Auto in den Urlaub. Herr Schmidt fährt mit konstanter Geschwindigkeit auf der Autobahn. Die Familie entscheidet sich bei einem Picknick-Platz zu halten. Nach der Pause ist die Autobahn überfüllt und die Fahrt macht keinen Spaß mehr.

Quelle des Items: Leichte Modifikation des Vera8-Items „Geschichte zur Graphik" des Institut für Qualitätsentwicklung im Bildungswesen. (2012). *Beispielaufgaben Mathematik Sek I*. Berlin: Autor. Verfügbar unter https://www.iqb.hu-berlin.de/vera/aufgaben/ma1

Blumenvase

In eine Blumenvase wird gleichmäßig Wasser eingelassen. Der Graph beschreibt die Höhe des Wasserstandes in Abhängigkeit von der Zeit.

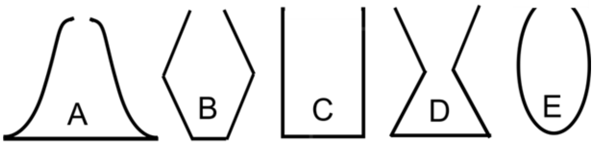

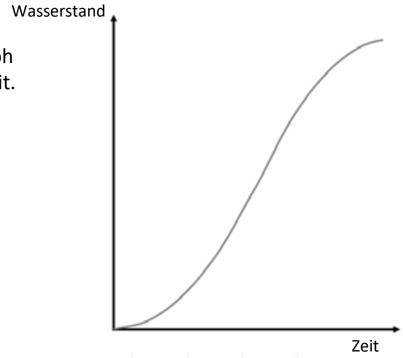

Welche Form hat die Vase ungefähr?

☐ A
☐ B
☐ C
☐ D
☐ E

Quelle des Items: Eigenentwicklung

Swimmingpool

In einen Swimmingpool mit dem abgebildeten Querschnitt wird mit einem Schlauch Wasser eingelassen. Aus dem Schlauch fließt das Wasser über den gesamten Zeitraum mit der gleichen Geschwindigkeit.

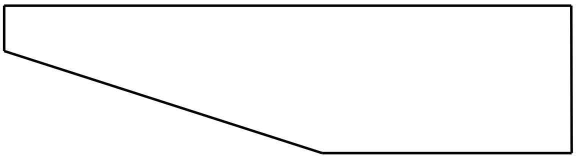

Zeichne in das Koordinatensystem ein, wie die Wasserhöhe im Swimmingpool im Verlauf der Zeit ansteigt.

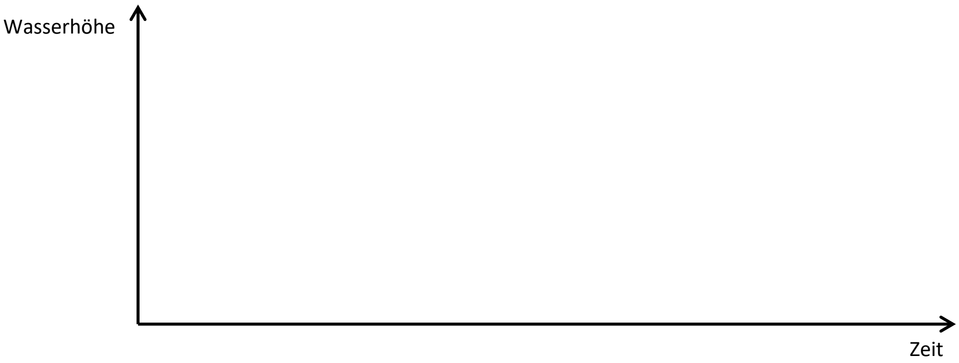

Quelle des Items: Eigenentwicklung

Fahrsteig

Nebenstehend ist ein Foto von einem Fahrsteig abgebildet. Das untenstehende Koordinatensystem zeigt den Vergleich zwischen „auf dem Fahrsteig gehen" und „auf dem Boden neben dem Fahrsteig gehen" an.

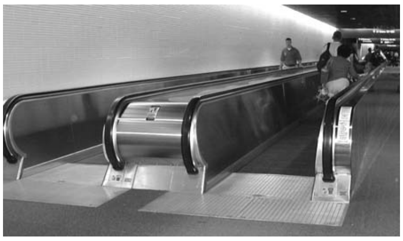

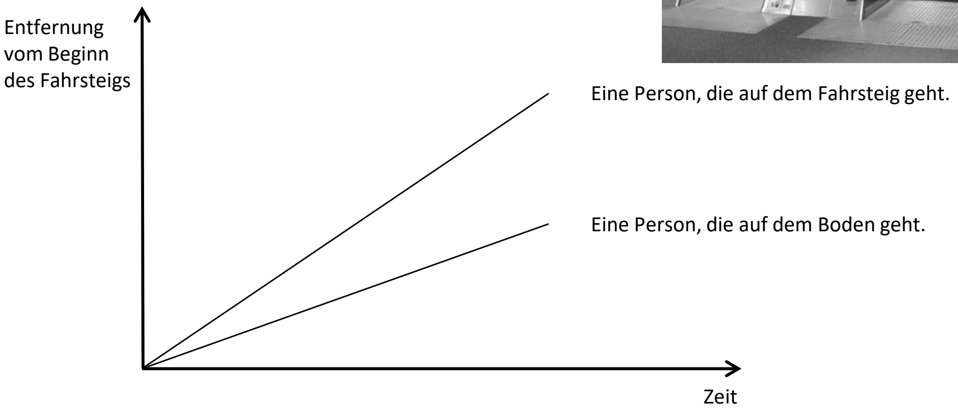

Nimm an, dass im obigen Graphen die beiden Personen ungefähr mit der gleichen Geschwindigkeit gehen. Zeichne in das Koordinatensystem eine weitere Linie für eine dritte Person ein, die auf dem Fahrsteig steht.

Quelle des Items: deutsche Übersetzung des Items M703 „Moving walkways" der Organisation for Economic Co-operation and Development. (2003). *The PISA 2003 Assessment Framework. Mathematics, Reading, Science and Problem Solving Knowledge and Skills.* Paris: OECD Publishing.

Laufen

Lisa läuft seit 2 Minuten mit konstanter Geschwindigkeit. Dann wird sie gleichmäßig schneller.

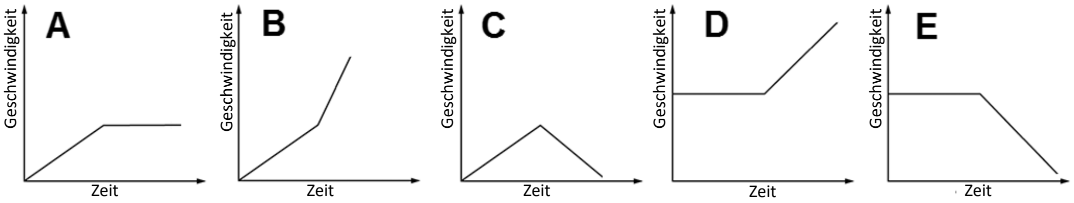

Welcher der oben abgebildeten Graphen zeigt, wie sich Lisas Geschwindigkeit mit der Zeit verändert?

☐ A
☐ B
☐ C
☐ D
☐ E

Quelle des Items: deutsche Übersetzung des Items mit der Question-ID 2011-8M9 #3 M170601 von U.S. Department of Education, Institute of Education Sciences, National Center for Education Statistics. (2011). *National Assessment of Educational Progress (NAEP) - 2011 Mathematics Assessment.* Washington, DC: Autor. Verfügbar unter https://nces.ed.gov/NationsReportCard/nqt/Search

Kugelvase

Nebenstehend ist ein Foto von einer kugelförmigen Vase abgebildet. In die Vase wird gleichmäßig Wasser eingefüllt. Zeichne in das untenstehende Koordinatensystem den Graphen für den Wasserstand im Verlauf der Zeit ein.

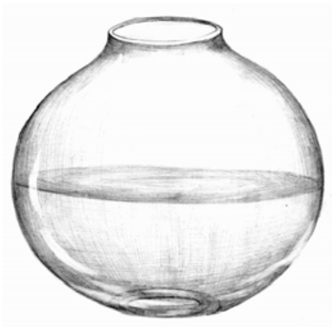

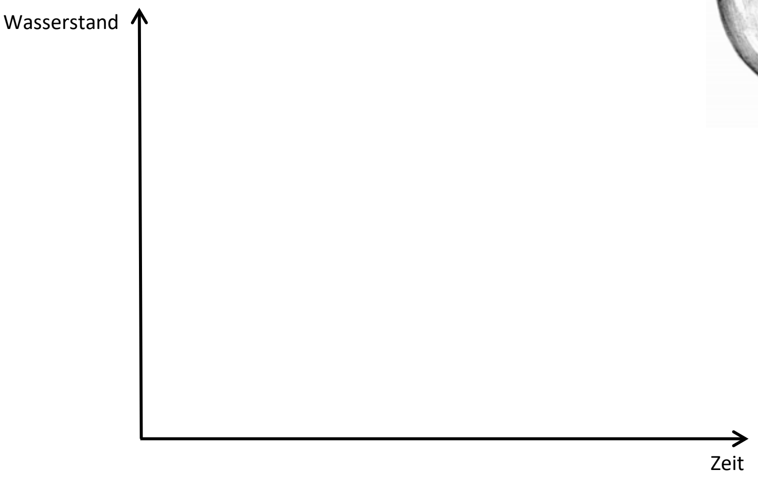

Quelle des Items: Eigenentwicklung

Fahrzeug

In der Abbildung ist der zurückgelegte Weg in Abhängigkeit von der Zeit von fünf verschiedenen Fahrzeugen abgebildet.

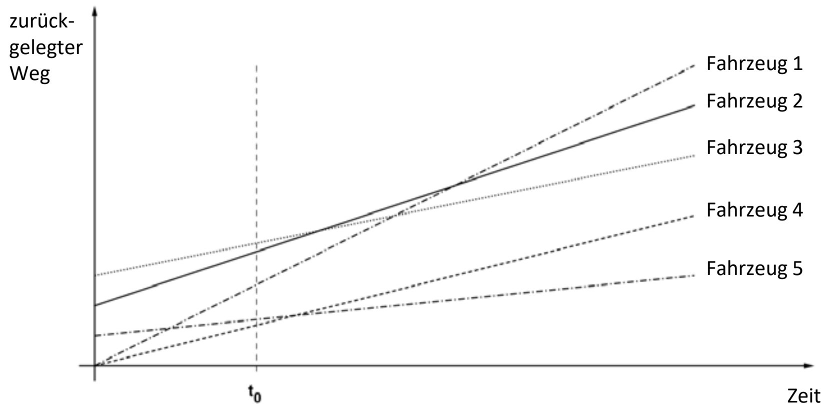

Welches Fahrzeug ist zum Zeitpunkt t_0 am schnellsten?

☐ Fahrzeug 1

☐ Fahrzeug 2

☐ Fahrzeug 3

☐ Fahrzeug 4

☐ Fahrzeug 5

Quelle des Items: Eigenentwicklung

Wachstum

Im Graphen ist das Größenwachstum von Max dargestellt.

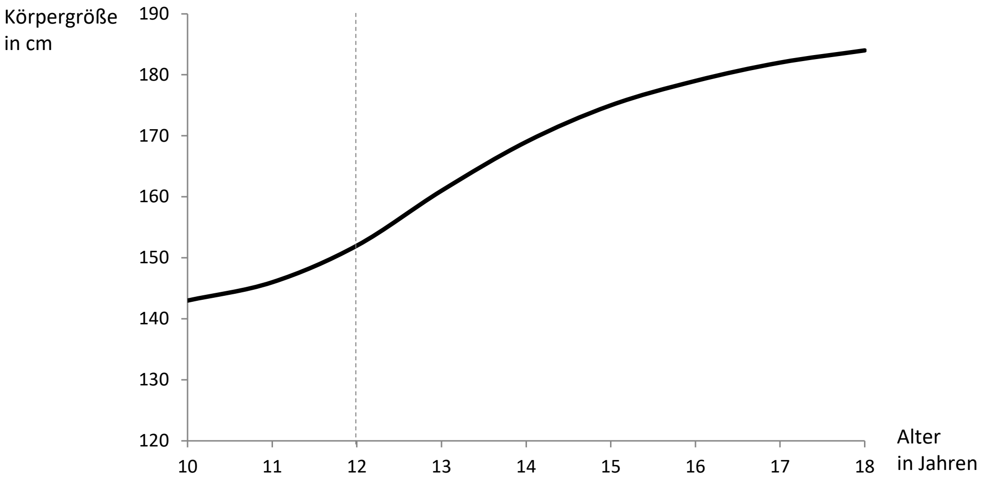

Maria ist mit 12 Jahren zwar kleiner als Max, wächst aber in diesem Alter erheblich schneller als Max. Zeichne in den Graphen ein, wie die Wachstumslinie für Maria aussehen könnte.

Quelle des Items: Eigenentwicklung

Skifahren

Der Skifahrer fährt einen Hang herunter. An der abgebildeten Stelle hat er bereits eine gewisse Geschwindigkeit erreicht.

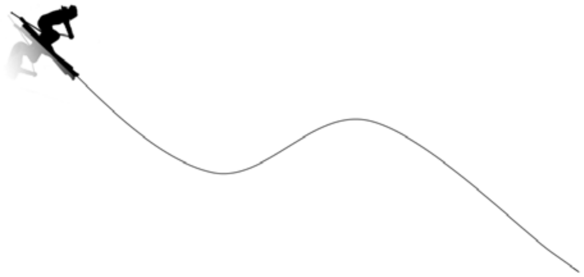

Skizziere in dem untenstehende Koordinatensysten wie sich die Geschwindigkeit des Skifahrers im Verla

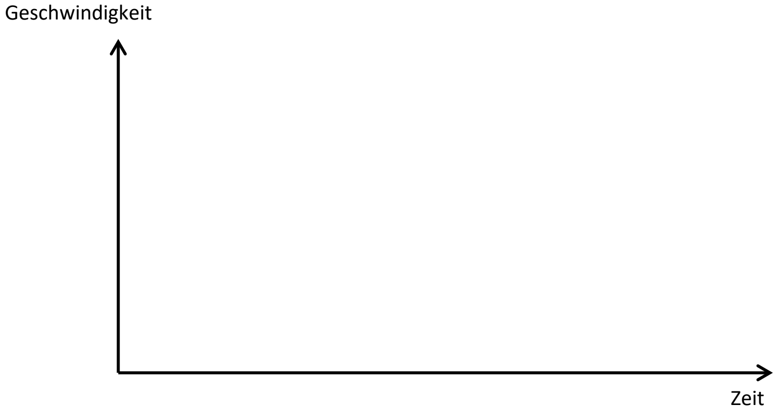

Quelle des Items: Leichte Modifikation eines Items aus Schlöglhofer, F. (2000). Vom Foto-Graph zum Funktions-Graph. *Mathematik lehren, 103,* 16–21.

Handytarif

Paul hat die Auswahl zwischen zwei Handytarifen. In dem untenstehende Koordinatensystem soll dargestellt werden, welche monatlichen Kosten in Abhängigkeit von den telefonierten Minuten entstehen. Zeichne dazu jeweils einen Graphen für Tarif A und einen Graphen für Tarif B ein.

Tarif A
Telefoniere so viel wie du möchtest und zahle immer nur 20 Euro! Die Super-Flatrate für junge Leute!

Tarif B
Keine monatliche Grundgebühr. Die ersten 50 Minuten kosten 10 Cent pro Minute, jede weitere Minute im Monat kostet 5 Cent pro Minute.

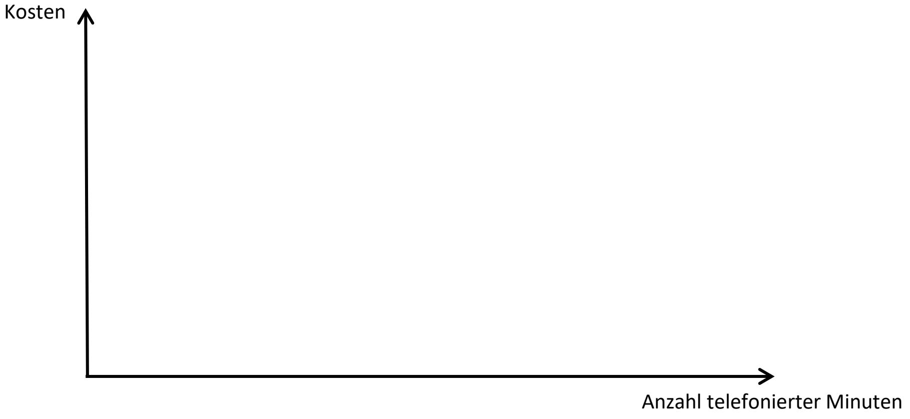

Quelle des Items: Eigenentwicklung

(Source: Rolfes, T. (2018). *Funktionales Denken: Empirische Ergebnisse zum Einfluss von statischen und dynamischen Repräsentationen. Anhang.* Landau: Autor. https://doi.org/10.13140/RG.2.2.26605.64485)
