# Supplementary material for: Learning the Concept of Function With Dynamic Visualizations
Source: Front Psychol. 2020 Apr 30;11:693. doi: 10.3389/fpsyg.2020.00693 (PMC7212367; doi:10.3389/fpsyg.2020.00693)
Supplement: Supplementary file 3 [file Data_Sheet_3.PDF]

Supplementary Material 3: Posttest Items

Aufgabe 1

Rechts ist ein rechtwinkliges Dreieck zu sehen, welches bei Punkt B den rechten Winkel hat.

Wie zuvor auch, liegt der Punkt P fest im Punkt A. Der Punkt Q kann jetzt nicht in einer Animation bewegt werden, sondern stelle dir in Gedanken vor, man würde den Punkt Q auf der Randlinie verschieben.

Unten ist ein Bild zu sehen. Wird die Sehne s am kürzesten, wenn der Punkt Q im Punkt R, S, T, U oder V liegt?

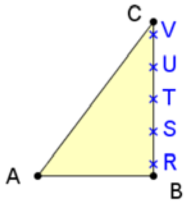

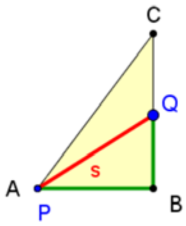

Wähle die zutreffende Antwort aus!

- ○ R
- ○ S
- ○ T
- ○ U
- ○ V

Weiter

Aufgabe 2

In welchen Bereichen ändert sich die Sehnenlänge nicht gleichmäßig, wenn sich der Punkt Q mit konstanter Geschwindigkeit auf der Randlinie bewegt?

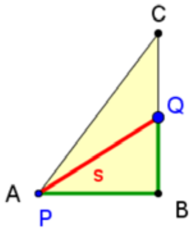

Wähle die zutreffende Antwort aus!

- ○ zwischen A & B und zwischen B & C
- ○ nur zwischen B & C
- ○ zwischen B & C und zwischen C & A
- ○ nur zwischen C & A
- ○ zwischen A & B und zwischen C & A

Weiter

Aufgabe 3

Der Punkt Q bewegt sich wieder mit konstanter Geschwindigkeit auf der Randlinie. Bei welchem der unten abgebildeten Punkte verändert sich die Länge der Sehne s am langsamsten?

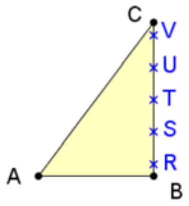

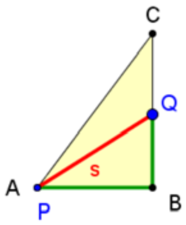

Wähle die zutreffende Antwort aus!

- R
- S
- T
- U
- V

Weiter

Aufgabe 4

Wie sieht für das rechtwinklige Dreieck der Gesamtgraph aus, wenn man die Sehnenlänge und den Weg in einem Koordinatensystem abträgt?

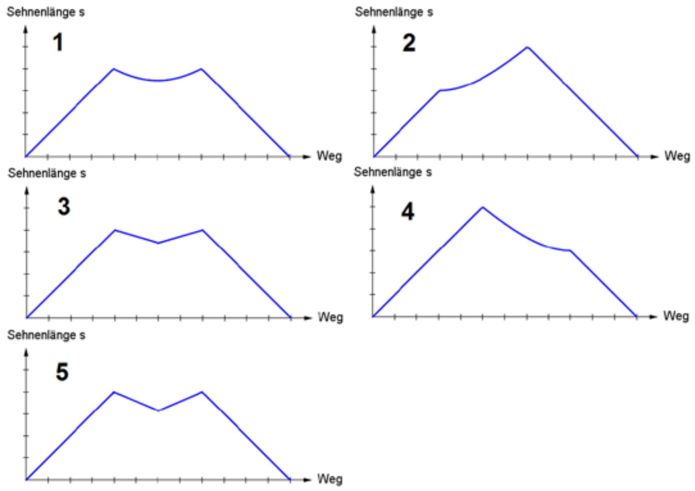

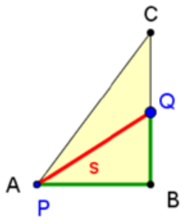

Wähle die zutreffende Antwort aus!

- Graph 1
- Graph 2
- Graph 3
- Graph 4
- Graph 5

Weiter

Aufgabe 5

Nun ist ein anderes rechtwinkliges Dreieck zu sehen. Auch hier liegt der Punkt P im Punkt A. Der Punkt Q bewegt sich wieder mit konstanter Geschwindigkeit auf der Randlinie.

Bei welchem der unten abgebildeten Punkte verändert sich die Länge der Sehne s am langsamsten?

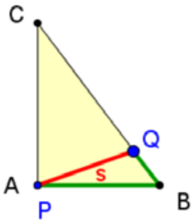

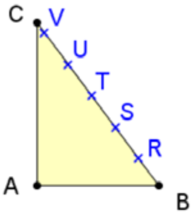

Wähle die zutreffende Antwort aus!

- R
- S
- T
- U
- V

Weiter

Aufgabe 6

Wie sieht für das rechtwinklige Dreieck der Gesamtgraph aus, wenn man die Sehnenlänge und den Weg in einem Koordinatensystem abträgt?

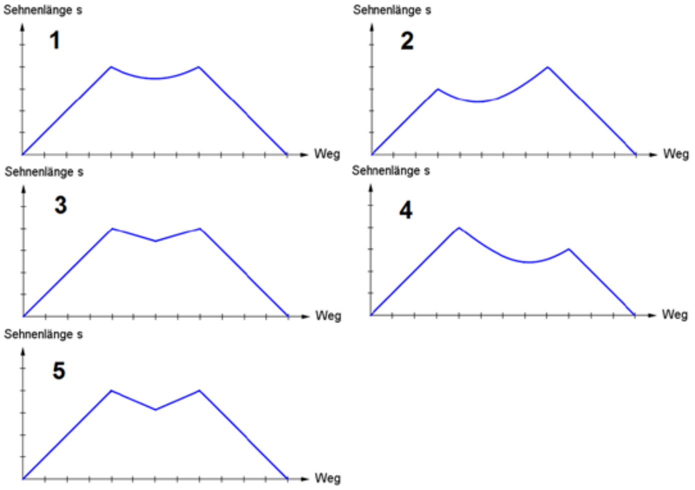

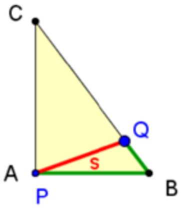

Wähle die zutreffende Antwort aus!

- Graph 1
- Graph 2
- Graph 3
- Graph 4
- Graph 5

Weiter

Aufgabe 7

Nun ist rechts ein senkrecht stehendes Rechteck zu sehen. Der Punkt P liegt wieder fest in Punkt A.

Der Punkt Q bewegt sich wieder mit konstanter Geschwindigkeit auf der Randlinie. Bei welchem Punkt muss er sich befinden, damit sich die Länge der Sehne am langsamsten verändert?

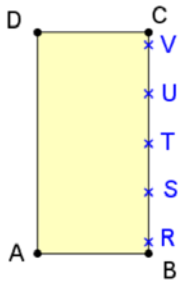

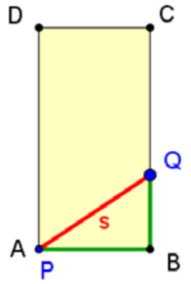

Wähle die zutreffende Antwort aus!

- R
- S
- T
- U
- V

Weiter

Aufgabe 8

In welchen Bereichen nimmt die Sehnenlänge gleichmäßig zu, wenn der Punkt Q wieder mit konstanter Geschwindigkeit die Randlinie durchwandert?

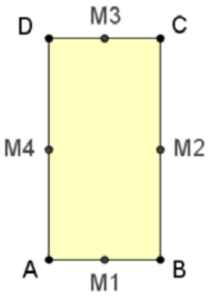

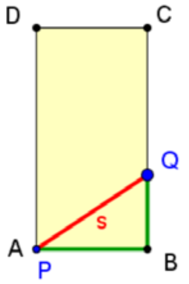

Wähle die zutreffende Antwort aus!

- zwischen M1 & B
- zwischen B & M2
- zwischen M2 & C
- zwischen M3 & D
- zwischen D & M4

Weiter

Aufgabe 9

Wie sieht der Gesamtgraph aus?

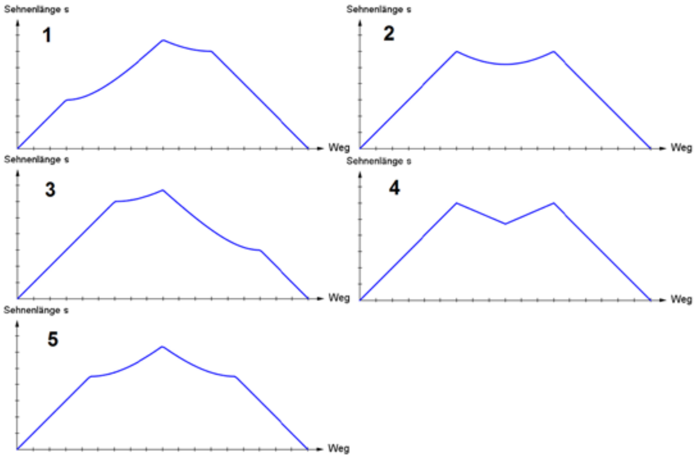

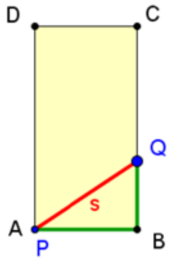

Wähle die zutreffende Antwort aus!

- ○ Graph 1
- ○ Graph 2
- ○ Graph 3
- ○ Graph 4
- ○ Graph 5

Weiter

Aufgabe 10

Nun ist rechts ein beliebiges Fünfeck zu sehen. Der Punkt P befindet sich erneut im Punkt A.

Der Punkt Q bewegt sich wieder mit konstanter Geschwindigkeit auf der Randlinie.

Bei welchem Punkt muss sich der Punkt Q befinden, damit sich die Länge der Sehne am langsamsten verändert?

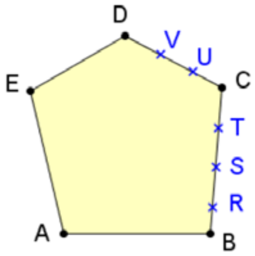

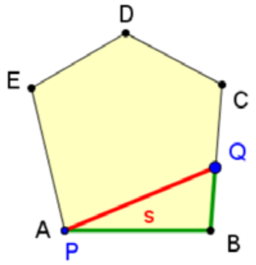

Wähle die zutreffende Antwort aus!

- ○ R
- ○ S
- ○ T
- ○ U
- ○ V

Weiter

Aufgabe 11

Konstante Geschwindigkeit von Punkt Q wieder vorausgesetzt: In welchen Bereichen nimmt die Sehnenlänge gleichmäßig ab?

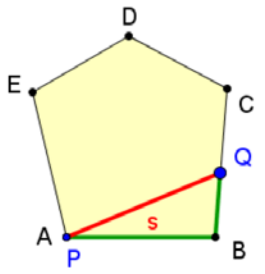

Wähle die zutreffende Antwort aus!

- zwischen A & B
- zwischen B & C
- zwischen C & D
- zwischen D & E
- zwischen E & A

Weiter

Aufgabe 12

Wie sieht der Gesamtgraph aus?

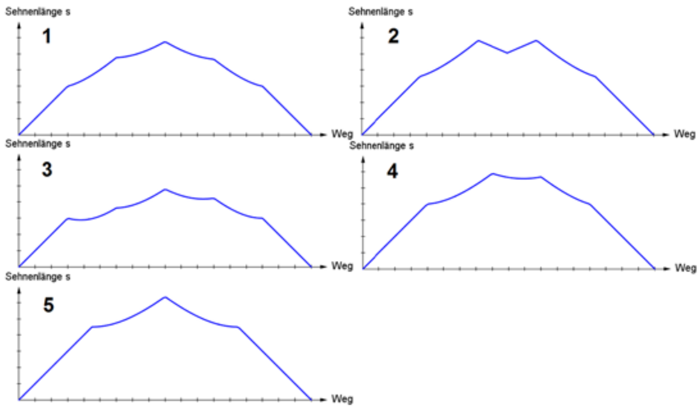

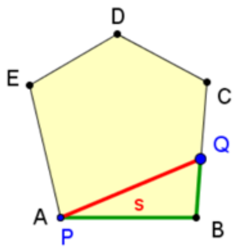

Wähle die zutreffende Antwort aus!

- Graph 1
- Graph 2
- Graph 3
- Graph 4
- Graph 5

Weiter

Aufgabe 13

Jetzt ist eine Raute zu sehen. Der Punkt P liegt wieder fest in Punkt A und der Punkt Q bewegt sich wieder mit konstanter Geschwindigkeit auf der Randlinie.

Bei welchem der unten abgebildeten Punkte verändert sich die Länge der Sehne s am langsamsten?

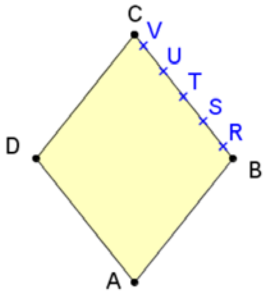

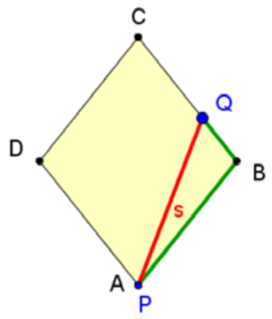

Wähle die zutreffende Antwort aus!

- ○ R
- ○ S
- ○ T
- ○ U
- ○ V

Weiter

Aufgabe 14

Wie sieht für die Raute der Gesamtgraph aus, wenn man die Sehnenlänge und den Weg in einem Koordinatensystem abträgt?

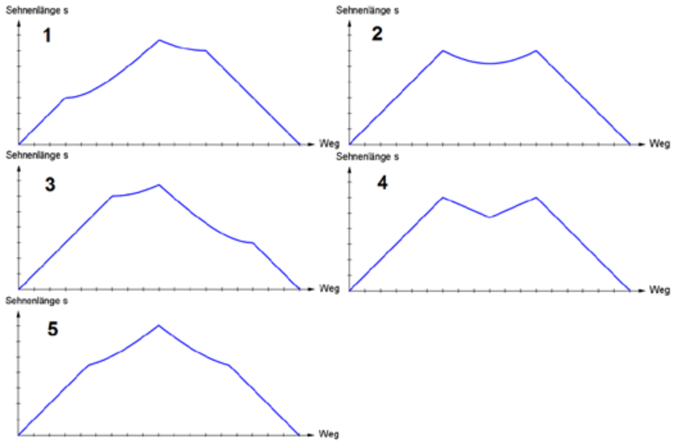

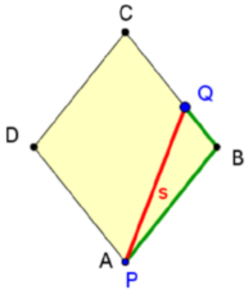

Wähle die zutreffende Antwort aus!

- ○ Graph 1
- ○ Graph 2
- ○ Graph 3
- ○ Graph 4
- ○ Graph 5

Weiter

Aufgabe 15

Rechts ist ein Gesamtgraph zu sehen. Überlege genau, welche Form die zugehörige Figur haben muss.

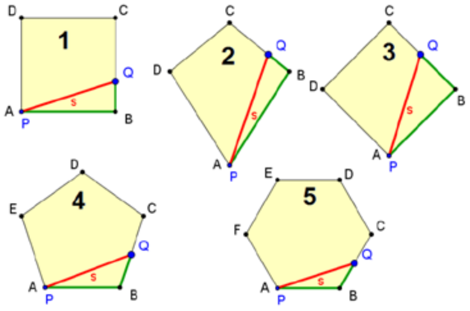

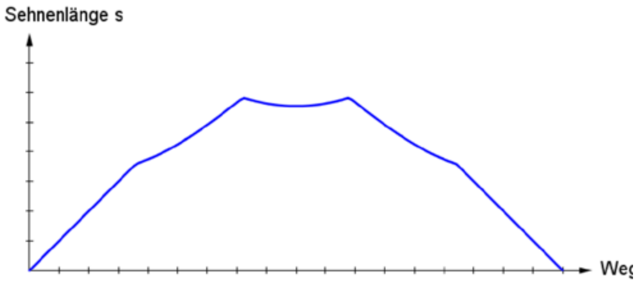

Wähle die zutreffende Antwort aus!

- ○ Form 1
- ○ Form 2
- ○ Form 3
- ○ Form 4
- ○ Form 5

Weiter

(Source: Rolfes, T. (2018). *Funktionales Denken: Empirische Ergebnisse zum Einfluss von statischen und dynamischen Repräsentationen. Anhang.* Landau: Autor. https://doi.org/10.13140/RG.2.2.26605.64485)
